# Genome-based identification of the CYP75 gene family in Orchidaceae and its expression patterns in *Cymbidium goeringii*

**DOI:** 10.3389/fpls.2023.1243828

**Published:** 2023-09-27

**Authors:** Yuanyuan Li, Xuewei Zhao, Meng-Meng Zhang, Xin He, Ye Huang, Sagheer Ahmad, Zhong-Jian Liu, Siren Lan

**Affiliations:** ^1^ Key Laboratory of National Forestry and Grassland Admini stration for Orchid Conservation and Utilization at College of Landscape Architecture and Art, Fujian Agriculture and Forestry University, Fuzhou, China; ^2^ College of Forestry, Fujian Agriculture and Forestry University, Fuzhou, China

**Keywords:** Orchidaceae, cytochrome P450, CYP75 gene family, expression analysis, *Cymbidium goeringii*

## Abstract

With a great diversity of species, Orchidaceae stands out as an essential component of plant biodiversity, making it a primary resource for studying angiosperms evolution and genomics. This study focuses on 13 published orchid genomes to identify and analyze the CYP75 gene family belonging to the cytochrome P450 superfamily, which is closely related to flavonoid biosynthetic enzymes and pigment regulation. We found 72 *CYP75*s in the 13 orchid genomes and further classified them into two classes: CYP75A and CYP75B subfamily, the former synthesizes blue anthocyanins, while the latter is involved in the production of red anthocyanins. Furthermore, the amount of *CYP75B*s (53/72) greatly exceeds the amount of *CYP75A*s (19/72) in orchids. Our findings suggest that *CYP75B* genes have a more important evolutionary role, as red plants are more common in nature than blue plants. We also discovered unique conserved motifs in each subfamily that serve as specific recognition features (motif 19 belong to CYP75A; motif 17 belong to CYP75B). Two diverse-colored varieties of *C. goeringii* were selected for qRT-PCR experiments. The expression of *CgCYP75B1* was significantly higher in the purple-red variant compared to the yellow-green variant, while *CgCYP75A1* showed no significant difference. Based on transcriptomic expression analysis, *CYP75B*s are more highly expressed than *CYP75A*s in floral organs, especially in colorful petals and lips. These results provide valuable information for future studies on *CYP75*s in orchids and other angiosperms.

## Introduction

CYP450 (cytochrome P450, also called CYP) is named because it can bind to ferrous carbon monoxide and has a peak light absorption value of 450 nm (Cederbaum, 2015). In 1962, “CYP450” was first named as a colored substance in the cell, and it exists as an enzyme (Omura and Sato, 1962). It is one of the largest protein superfamilies in nature that almost exists in all prokaryotes and eukaryotes, especially with a large number in plants (Kiani and Jabeen, 2019; Li and Wei, 2020). They are a series of self-oxidating heme enzymes involved in various primary and secondary metabolic activities in plants and participate in biosynthetic pathways in flavonoids, aliphatic acids, sterols, phytohormones, terpenes, lignins and other biomolecules (Schuler and Werck-Reichhart, 2003; Kumar et al., 2014). In general, *CYP*s can be divided into a gene family with amino acid sequence identity greater than 40% and a subfamily with amino acid sequence identity greater than 55% (Nelson et al., 1996). CYP71−CYP99 and CYP701−CYP999 and found in plants (Liu et al., 2020). *CYP75* is a crucial member that regulates flavonoid biosynthesis that determines pigmentation of plant tissues (Zhang et al., 2019). CYP75 gene family includes two subfamilies, CYP75A and CYP75B, regulating two key enzymes in the anthocyanin synthesis pathways, including Flavonoid 3’,5’-Hydroxylase (F3’5’H) and Flavonoid 3’-Hydroxylase (F3’H) which are precursors of blue and red anthocyanins, respectively (Tanaka and Brugliera, 2013).

Recent studies have discussed the function of *CYP75*s in several plants. They were first identified in the *Petunia hybrida*, wherein the introduction of clonal *F3’H* cDNA caused an unusual pink color of petunia and their pollen produced paeoniflorin (Holton et al., 1993; Brugliera et al., 1999). In *Arabidopsis thaliana*, a *TT7* gene was identified, which could encode F3’H and its function was validated in flavonoid and anthocyanins synthesis (Schoenbohm et al., 2000). The *sF3’H1* of *Glycine max* is mainly responsible for the alteration of pubescent color from brown to gray (Toda et al., 2002). In *Vitis vinifera*, *CYP75* genes encoding F3’H and F3’5’H are highly expressed in all tissues of the plant, especially in the epidermis of mature red berries that mainly synthesize anthocyanins (Falginella et al., 2010). In *Solanum lycopersicum*, *CYP75A31* encodes a F3’5H, which accepts flavones, flavanones, dihydroflavonols and flavonols as substrates (Olsen et al., 2010). In *Epimedium sagittatum*, *EsF3’H* and *EsF3’5H* genes are highly expressed in colored tissues and their expressions are positively correlated with the pattern of anthocyanin accumulation in leaves (Huang et al., 2012). The *CsF3’5’H* in *Camellia sinensis* acts as a key agent controlling trihydroxyflavone-3-alcohol synthesis and effectively converts 4’-hydroxylated flavonoids into 3’4’5’- and/or 3’4’-hydroxylated products (Wang et al., 2014). The *F3’H-1* and *F3’H-2* in *Hordeum vulgare* regulates the accumulation of magenta pigments in peels and stems, respectively, and the *F3’5’H-1* is closely related to the accumulation of blue pigments in the barley grain paste layers (Vikhorev et al., 2019). With the continuous advancement of molecular experimental research, it is now possible to change plants color by controlling regulatory genes, such as *CYP75*. For example, the up-regulated of *CYP75A* and *CYP75B* genes which encode a critical dihydroflavonol 4-reductase (DFR) in *Dendrobium officinale*, may lead to anthocyanins accumulation, promoting color change from green to red (Niu et al., 2021). However, the studies confirmed that *CYP75B* can code F3’H instead of *CYP75A*, and some species in Asteraceae have F3’5’H with the same amino acid sequences as F3’H which suggests that the F3’5’H in these plants is regulated by *CYP75B* instead of *CYP75A* (Seitz et al., 2006). Similar to *Callistephus chinensis* (F3’5’H: *CYP75B5*), *Osteospermum hybrida* (F3’5’H: *CYP75B17*)and *Pericallis cruenta* (F3’5’H: *CYP75B18*), they all lost *CYP75A* genes to code F3’5’H then reacquired by duplication and neofunctionalization of *CYP75B* genes (Seitz et al., 2006). This is further proved by partial amino acid exchanges between F3’5’H and F3’H (Seitz et al., 2007).

Orchidaceae is one of the largest monocotyledon families, boasting significant ornamental and economic importance due to its diverse range of colors and distinctive flower shapes (Li et al., 2021). There are more than 28,000 species and 850 genera in Orchidaceae, represents approximately 10% of all flowering plants worldwide and has the largest number of species (Chase et al., 2015). Orchids are remarkable for shedding light on plant evolution, with more complete orchid genomes now available, researchers have gained significant insight into the genetic foundations of orchid biology (Zhang et al., 2021a). Extensive research has been conducted on *CYP75*s in model plants, but there is currently limited knowledge about the characteristics of these genes in the Orchidaceae. In this study, we performed genome-wide identification, classification, characterization, and expression pattern analysis of *CYP75*s in 13 orchids, representing four subfamilies of Orchidaceae with considerable diversity to elucidate the evolution of *CYP75*s in orchids. The findings could provide new insights into the underlying mechanisms that drive the evolution and diversification of organ morphology in orchids and other flowering plants.

## Materials and methods

### Data sources

To investigate the features of the CYP75 gene family in Orchidaceae, 13 orchids with completed whole-genome sequencing were selected. They include *C. goeringii* (Chung et al., 2021; Sun et al., 2021), *C. sinense* (Yang et al., 2021), *C. ensifolium* (Ai et al., 2021), *Gastrodia elata* (Yuan et al., 2018; Xu et al., 2021), *D. catenatum* (Zhang et al., 2016; Niu et al., 2021), *D. chrysotoxum* (Zhang et al., 2021b), *D. huoshanense* (Han et al., 2020), *Phalaenopsis aphrodite* (Chao et al., 2018), *P. equestris* (Cai et al., 2015) of Epidendroideae, *Platanthera guangdongensis* (Li et al., 2022), *Pl. zijinensis* (Li et al., 2022) of Orchidoideae, *Vanilla planifolia* (Hasing et al., 2020) of Vanilloideae, *Apostasia shenzhenica* (Zhang et al., 2017) of Apostasioideae. The genome download urls for 13 orchids can be found in **Table S1**. And four CYP75A (AUB13331.1 from *Horgeum vulgare*; ABI95365.1 and AAZ79451.1 from *D. hybrid*; AEB96145.1 from *D. moniliforme*) and four CYP75B (BAJ93256.1 from *H. vulgare*; AT5G07990.1 from *A. thaliana*; XP015613041.1 from *Oryza sativa*; AF155332.1 from *Petunia hybrida*) proteins were downloaded from the NCBI (https://www.ncbi.nlm.nih.gov/). All the protein sequences can be found in the **Table S2**.

### Identification and physicochemical properties of *CYP75*s

**Figure 1:**
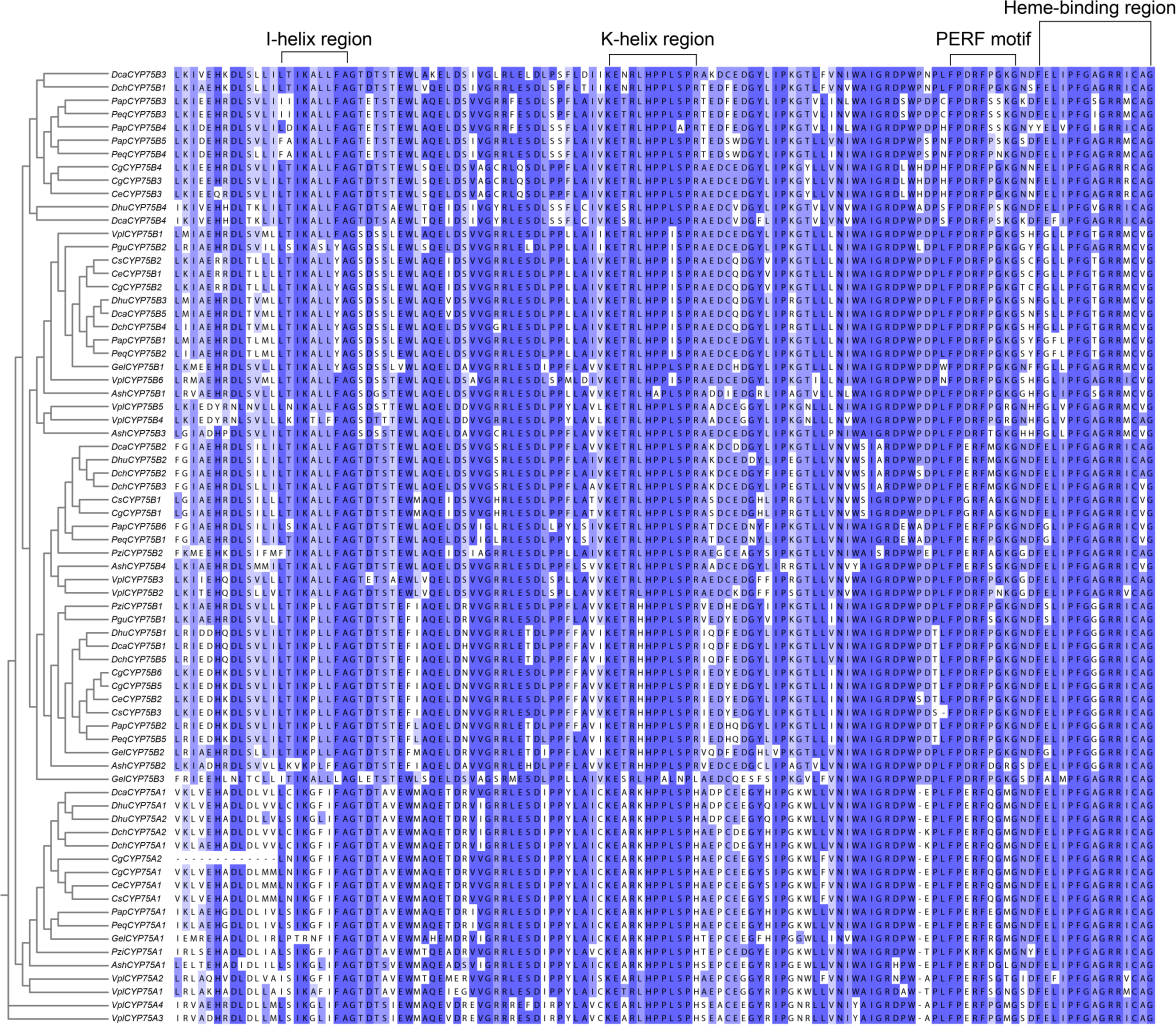
The typical motifs in the CYP protein amino acid sequences. Multiple sequence alignments were constructed by MAFFT, and Jalview software was used to visualize the sequences (Troshin et al., 2011; Rozewicki et al., 2019).

It is complicated to identify *CYP*s due to their extremely high sequence conservation (Mizutani and Ohta, 2010; Babu et al., 2013; Kumar et al., 2014; Reddy et al., 2014). Therefore, eight CYP75 proteins were used as queries to perform a blast search (built-in TBtools; Chen et al., 2018) against 13 orchid genomes with an E-value threshold of e^-20^ and a requirement for amino acid sequence similarity greater than 50% (Falginella et al., 2010). Pfam verification was performed on all protein sequences in the blast results. The CYP domains (PF00067) built on the hidden Markov model were downloaded from Sanger center (http://pfam.sanger.ac.uk/). The identification of all alternative orchid CYPs was conducted using the Hmmsearch program (built-in Tbtools; Chen et al., 2018). The subsequent screening study included all protein sequences containing CYP domains. To facilitate identification, 14 A*. thaliana* CYP proteins (AtCYP71A12, AtCYP71A13, AtCYP71B15, AtCYP73A5, AtCYP76C1, AtCYP77B1, AtCYP77A6, AtCYP78A10, AtCYP82G1, AtCYP84A1, AtCYP89A2, AtCYP98A3, AtCYP701A3, AtCYP706A1) belonging to subfamilies other than CYP75 were also downloaded from the TAIR (https://www.arabidopsis.org/). Then, ML phylogenetic trees were constructed for further screening of *CYP75* genes based on all *CYP* genes of each orchid and other species. The phylogenetic analysis was conducted using the maximum likelihood (ML) approach, and the ML tree was constructed using the RAxML on the CIPRES Science Gateway web server (RAxML-HPC2 on XSEDE; Miller et al., 2015) with 1,000 bootstrap iterations. The phylogenetic tree of *CYP*s for each orchid is detailed in the **Figure S1**. The target genes were clustered with eight *CYP75* query genes. Ultimately, we aligned all candidate CYP75 protein sequences of 13 orchids and further eliminated the incomplete gene annotation sequences (**Figure 1**). The completed protein sequences of orchid *CYP75*s can be found in **Table S2**. The physicochemical properties of CYP75 proteins were predicted by ExPASy database (https://www.expasy.org/) (Artimo et al., 2012). Subcellular localization was predicted by Plant-mPloc (http://www.csbio.sjtu.edu.cn/bioinf/plant-multi/#) (Chou and Shen, 2010).

### Phylogenetic analyses

We performed multiple sequence alignment of CYP75 proteins from 13 orchids and other species using MEGA 7.0 software (Kumar et al., 2016). The alignment sequences selected with the ClustalW program, Gap Opening and Gap Extend, are 15 and 6.66, respectively; the DNA Weight Matrix selection is the IUB; other values keep the default. The phylogenetic analysis was conducted using the maximum likelihood (ML) approach, and the ML tree was constructed using the RAxML on the CIPRES Science Gateway web server (RAxML-HPC2 on XSEDE; Miller et al., 2015) with 1,000 bootstrap iterations.The best model (JTT+I+G4) was determined by using the Akaike Information Criterion (AIC) and the Bayesian Information Criterion (BIC) based on modeltest-ng-0.1.3 (Darriba et al., 2019). Keep other settings as default. The output phylogenetic tree file was polished using Evolview (http://www.evolgenius.info/evolview/) (He et al., 2016).

### Motif and gene structure analysis

To illustrate the variations of motifs among orchid CYP75 proteins, the MEME motif search tool was employed to detect the conserved motifs in all CYP75 proteins (Bailey et al., 2009). The MEME parameters were optimized as follows: the maximum number of motifs found was set to 20, and the optimal motif width ranged from six to 50 bases. GSDS6 (http://gsds.gao-lab.org/) (Hu et al., 2015) was used for analyzing structure. The protein motifs and gene structures of *CYP75*s were visualized with TBtools (Chen et al., 2018).

### Collinearity and location analysis on chromosome

Utilizing chromosome-level genome assemblies of *C. goeringii*, *C. ensifolium*, and *D. chrysotoxum*, genomic FASTA files were merged pairwise to generate a database for BLASTp queries. The merged BLAST files and modified GFF3 files for each species were analyzed using MCscanX (Wang et al., 2012) to identify collinear blocks of *CYP75* genes between *C. goeringii* and *C. ensifolium*, *C. goeringii* and *D. chrysotoxum*. The dual synteny plotter tool of MCscanX (JCVI kit) was utilized for visualization of the results pertaining to collinearity.

We selected six orchids with chromosome-level genomes to analyze the distribution of *CYP75* genes on their chromosomes. According to the genome data and annotation data of *C. goeringii*, *C. ensifolium*, *D. huoshanense*, *D. chrysotoxum*, *Pl. guangdongensis* and *Pl. zijinensis*, the chromosomal localization of five orchid *CYP75* genes were visualized through TBtools (Chen et al., 2018).

### Prediction of *Cis*-acting elements

A total of 2,000bp upstream and downstream of *CgCYP75*s was extracted via TBtools, respectively (Chen et al., 2018). The online software PlantCARE (http://bioinformatics.psb.ugent.be/webtools/plantcare/html/; Lescot, 2002) was used to identify and annotate the *cis*-acting elements found in the upstream and downstream regions. *Cis*-acting element number and response function were visualized using TBtools (Chen et al., 2018).

### Transcriptome data and expression analysis

For transcriptome analysis, RSEM (Li and Dewey, 2011) was utilized to quantify transcripts and compute the fragment per kilobase of transcript per million mapped reads (FPKM) score for every gene. Expression heatmaps were produced using TBtools with the FPKM matrix (Chen et al., 2018). To confirm the expression patterns of the *CYP75*s, sepals, petals, labellums (lips), and gynostemiums were sampled from yellow-green *C. goeringii* (‘YG’) and purple-red *C. goeringii* (‘PR’), respectively, these were grown at Fujian Agriculture and Forestry University for quantitative real-time PCR (qRT-PCR) experiment. Each tissue type was sampled in three replicates. Total RNA of these tissues was extracted using the FastPure Plant Total RNA Isolation Kit (Vazyme Biotech Co., Ltd., Nanjing, China). First-strand DNA was synthesized with TransScript® All-in-One First-Strand cDNA Synthesis SuperMix for quantitative PCR (TransGen Biotech, Beijing, China). Premier 5 software was used to design primers for candidate genes and internal reference genes for qRT-PCR (Zhao et al., 2022). Gene-specific primers for two chosen genes and their corresponding internal control genes are presented in **Table S3**. The qRT-PCR was performed to verify the specific expression of *CgCYP75A1* and *CgCYP75B1* in the floral organs of ‘YG’ and ‘PR’ *C. goeringii*. All experiments were conducted in triplicate, with each run consisting of three technical replicates. The relative expression of genes was determined using the 2^-ΔΔCT^ method (Wang et al., 2021; Zhao et al., 2023).

### Gene ontology analysis

EggNOG-mapper v2 (http://eggnog-mapper.embl.de/) was used to perform a search against the eggNOG5.0 database for gene ontology (GO) functional annotation (Huerta-Cepas et al., 2019). The prediction of orthology was conducted through sequence alignment, while applying bit-score or E-value filtering to improve the quality of orthology assignments. Functional classification was attained by associating the GO annotation terms with the proteins involved in established biological processes. The results of the GO analysis are available in the **Table S7** and visualized using Tbtools (Chen et al., 2018).

## Results

### Identification and protein features of orchid *CYP75*s

A total of 72 *CYP75*s were identified from 13 orchids, with the number of *CYP75*s in each orchid ranging from two to ten (**Table 1**). Our study reveals that CYP75B subfamily has a significantly higher number (53/72) compared to CYP75A subfamily (19/72). Each of the 13 orchids contains one−four *CYP75A* genes and between two−six *CYP75B* genes. Among them, *V. planifolia* has the largest number of *CYP75* genes, with a total of ten (four *CYP75A* and six *CYP75B*), while *Pl. guangdongensis* has the smallest number with only two *CYP75B* genes and lacks *CYP75A* gene.

**Table 1:** A list of *CYP75* genes in 13 Orchids, their characteristics, and subcellular localization of proteins.

Species	Gene ID	Name	AA^a^(aa)	pI^b^	Mw^c^(kDa)	II^d^	AI^e^	GRAVY^f^	Localization^g^
*P. aphrodite*	*PAXXG087010*	*PapCYP75A1*	506	9.14	56.74	49.87	99.05	-0.069	Endoplasmic reticulum
	*PAXXG006510*	*PapCYP75B1*	551	6.69	61.37	39.40	97.64	-0.056	Endoplasmic reticulum
	*PAXXG079820*	*PapCYP75B2*	513	6.54	57.33	44.20	101.15	-0.123	Endoplasmic reticulum
	*PAXXG116530*	*PapCYP75B3*	506	7.73	56.38	37.35	103.70	0.021	Endoplasmic reticulum
	*PAXXG116560*	*PapCYP75B4*	503	6.68	55.90	38.79	102.45	-0.004	Endoplasmic reticulum
	*PAXXG116590*	*PapCYP75B5*	269	6.14	30.49	45.01	93.57	-0.138	Endoplasmic reticulum
	*PAXXG172030*	*PapCYP75B6*	522	6.62	57.28	32.68	101.65	0.042	Endoplasmic reticulum
*P. equestris*	*Peq013982*	*PeqCYP75A1*	491	7.65	54.77	43.57	94.75	-0.099	Endoplasmic reticulum
	*Peq002928*	*PeqCYP75B1*	489	6.69	53.89	34.32	99.12	-0.038	Endoplasmic reticulum
	*Peq006329*	*PeqCYP75B2*	531	6.62	59.14	39.48	98.93	-0.053	Endoplasmic reticulum
	*Peq008143*	*PeqCYP75B3*	271	5.59	30.79	36.80	98.97	-0.084	Endoplasmic reticulum
	*Peq008146*	*PeqCYP75B4*	511	8.39	57.02	39.79	100.80	0.015	Endoplasmic reticulum
	*Peq013868*	*PeqCYP75B5*	516	6.54	57.48	45.36	102.07	-0.111	Endoplasmic reticulum
*C. goeringii*	*GL07540*	*CgCYP75A1*	503	8.75	55.82	48.29	98.53	-0.030	Endoplasmic reticulum
	*GL10771*	*CgCYP75A2*	235	4.83	26.10	41.03	93.83	-0.076	Endoplasmic reticulum
	*GL07339*	*CgCYP75B1*	533	9.24	58.66	36.56	98.26	-0.042	Endoplasmic reticulum
	*GL13941*	*CgCYP75B2*	292	5.70	32.28	35.72	102.91	-0.092	Endoplasmic reticulum
	*GL26796*	*CgCYP75B3*	507	8.19	55.97	39.78	101.60	0.017	Endoplasmic reticulum
	*GL26797*	*CgCYP75B4*	272	5.99	30.47	39.98	96.18	-0.153	Endoplasmic reticulum
	*GL27961*	*CgCYP75B5*	517	6.91	57.31	46.28	103.23	-0.048	Endoplasmic reticulum
	*GL27962*	*CgCYP75B6*	290	5.63	32.20	41.23	96.59	-0.192	Endoplasmic reticulum
*C. ensifolium*	*JL017476*	*CeCYP75A1*	503	8.75	55.83	49.33	98.73	-0.026	Endoplasmic reticulum
	*JL011638*	*CeCYP75B1*	519	7.13	57.69	38.75	103.03	0.022	Endoplasmic reticulum
	*JL016547*	*CeCYP75B2*	517	6.94	57.38	45.00	104.16	-0.036	Endoplasmic reticulum
	*JL021317*	*CeCYP75B3*	523	8.41	57.82	39.78	100.36	-0.021	Endoplasmic reticulum
*C. sinense*	*Mol022332*	*CsCYP75A1*	503	8.64	55.95	48.61	98.33	-0.040	Endoplasmic reticulum
	*Mol004997*	*CsCYP75B1*	530	9.24	58.30	35.56	96.23	-0.070	Endoplasmic reticulum
	*Mol005994*	*CsCYP75B2*	531	8.47	58.95	38.51	102.17	-0.014	Endoplasmic reticulum
	*Mol021869*	*CsCYP75B3*	515	6.75	57.14	43.98	104.19	-0.036	Endoplasmic reticulum
*V. planifolia*	*Vpla_KAG0447236.1*	*VplCYP75A1*	507	8.45	56.24	43.53	100.89	0.053	Endoplasmic reticulum
	*Vpla_KAG0447237.1*	*VplCYP75A2*	270	5.13	29.74	32.81	91.85	-0.002	Endoplasmic reticulum
	*Vpla_KAG0495957.1*	*VplCYP75A3*	435	6.11	48.33	43.07	88.85	-0.116	Endoplasmic reticulum
	*Vpla_KAG0496007.1*	*VplCYP75A4*	503	8.10	55.92	47.05	93.70	-0.031	Endoplasmic reticulum
	*Vpla_KAG0451884.1*	*VplCYP75B1*	523	6.48	58.05	35.05	101.99	-0.030	Endoplasmic reticulum
	*Vpla_KAG0455627.1*	*VplCYP75B2*	506	7.00	56.21	36.62	98.08	-0.043	Endoplasmic reticulum
	*Vpla_KAG0455628.1*	*VplCYP75B3*	501	8.51	55.63	38.04	102.38	0.025	Endoplasmic reticulum
	*Vpla_KAG0466647.1*	*VplCYP75B4*	533	8.70	59.75	41.10	91.82	-0.238	Endoplasmic reticulum
	*Vpla_KAG0466648.1*	*VplCYP75B5*	481	8.58	53.86	38.34	93.06	-0.289	Endoplasmic reticulum
	*Vpla_KAG0497903.1*	*VplCYP75B6*	542	6.63	60.13	38.66	99.48	-0.052	Endoplasmic reticulum
*D. chrysotoxum*	*Maker09736*	*DchCYP75A1*	504	8.59	55.84	47.46	99.09	-0.037	Endoplasmic reticulum
	*Maker93966*	*DchCYP75A2*	504	8.39	55.86	47.46	99.27	-0.023	Endoplasmic reticulum
	*Maker59722*	*DchCYP75B1*	531	8.12	59.23	41.61	105.63	-0.019	Endoplasmic reticulum
	*Maker83891*	*DchCYP75B2*	519	6.75	57.49	43.90	100.39	-0.054	Endoplasmic reticulum
	*Maker109076*	*DchCYP75B3*	668	8.79	76.21	31.78	100.70	-0.086	Endoplasmic reticulum
	*Maker118630*	*DchCYP75B4*	552	7.28	61.45	37.57	94.71	-0.121	Endoplasmic reticulum
	*Maker118636*	*DchCYP75B5*	518	7.28	57.17	39.23	98.82	-0.027	Endoplasmic reticulum
*D. catenatum*	*Dca000941*	*DcaCYP75A1*	504	8.08	55.84	43.51	100.26	0.008	Endoplasmic reticulum
	*Dca000430*	*DcaCYP75B1*	523	7.80	57.90	40.36	101.68	-0.029	Endoplasmic reticulum
	*Dca008242*	*DcaCYP75B2*	512	6.86	56.69	31.44	98.11	-0.036	Endoplasmic reticulum
	*Dca013687*	*DcaCYP75B3*	525	7.75	59.56	39.05	103.07	-0.067	Endoplasmic reticulum
	*Dca013688*	*DcaCYP75B4*	512	8.04	57.41	39.50	103.03	0.006	Endoplasmic reticulum
	*Dca020470*	*DcaCYP75B5*	539	8.12	60.32	38.82	105.14	-0.027	Endoplasmic reticulum
*D. huoshanense*	*Dhu000016471*	*DhuCYP75A1*	504	7.63	55.75	43.84	99.48	-0.002	Endoplasmic reticulum
	*Dhu000016482*	*DhuCYP75A2*	504	8.66	55.83	45.45	99.70	-0.012	Endoplasmic reticulum
	*Dhu000012876*	*DhuCYP75B1*	521	7.29	57.71	40.21	101.69	-0.030	Endoplasmic reticulum
	*Dhu000016330*	*DhuCYP75B2*	517	6.31	57.35	33.97	96.79	-0.052	Endoplasmic reticulum
	*Dhu000019542*	*DhuCYP75B3*	539	8.39	60.33	38.66	104.42	-0.040	Endoplasmic reticulum
	*Dhu000020018*	*DhuCYP75B4*	531	8.12	59.23	41.61	105.63	-0.019	Endoplasmic reticulum
*Pl. guangdongensis*	*PGU007087*	*PguCYP75B1*	520	8.62	57.76	40.37	98.65	-0.086	Endoplasmic reticulum
	*PGU010950*	*PguCYP75B2*	522	8.96	58.06	52.92	101.69	-0.071	Endoplasmic reticulum
*Pl. zijinensis*	*PZI001224*	*PziCYP75A1*	504	9.41	55.86	47.87	101.61	0.031	Endoplasmic reticulum
	*PZI012105*	*PziCYP75B1*	520	8.32	57.64	40.20	99.04	-0.077	Endoplasmic reticulum
	*PZI015225*	*PziCYP75B2*	514	8.80	56.46	43.19	94.36	-0.047	Endoplasmic reticulum
*A. shenzhenica*	*Ash019093*	*AshCYP75A1*	508	6.63	56.67	47.90	98.17	-0.026	Endoplasmic reticulum
	*Ash001251*	*AshCYP75B1*	521	7.71	57.18	34.43	100.56	-0.016	Endoplasmic reticulum
	*Ash004102*	*AshCYP75B2*	520	7.26	57.78	42.60	95.44	-0.061	Endoplasmic reticulum
	*Ash007554*	*AshCYP75B3*	520	7.31	56.94	46.91	98.71	-0.036	Endoplasmic reticulum
	*Ash015225*	*AshCYP75B4*	508	6.22	56.16	45.84	95.61	-0.011	Endoplasmic reticulum
*G. elata*	*Gel004675*	*GelCYP75A1*	514	7.14	57.25	43.06	101.01	-0.064	Endoplasmic reticulum
	*Gel010620*	*GelCYP75B1*	512	8.79	56.24	49.58	96.45	-0.081	Endoplasmic reticulum
	*Gel016567*	*GelCYP75B2*	517	9.26	57.50	47.94	100.97	-0.064	Endoplasmic reticulum
	*Gel005429*	*GelCYP75B3*	504	8.37	56.27	42.41	102.64	0.013	Endoplasmic reticulum

AA^a^, Amino acid number; pI^b^, Theoretical isoelectric point; Mw^c^ (kDa), Molecular weight; II^d^, Instability index; AI^e^, Aliphatic index; GRAVY^f^, Grand average of hydrophobicity; Localization^g^, Subcellular localization predicted by Plant-mPloc (Chou and Shen, 2010).

The 72 CYP75 protein sequences range from 235−668 amino acids, with a mean of 493. The molecular weight ranges from 26.10−76.21 kDa, with a mean of 54.80 kDa. Around 88.89% (64/72) of the CYP75 proteins have high isoelectric points (pI>7), with an average of 7.57. The average instability index (II) is 41.39, and 39 CYP75 proteins are below this index, indicating good protein stability. The average aliphatic index (AI) for the 72 CYP75 proteins is 99.40, indicating high thermal stability. Moreover, the calculated mean hydrophilic index (GRAVY) of CYP75 proteins in all orchids is negative, indicating a high degree of hydrophilicity. All CYP75 proteins are localized within the endoplasmic reticulum (ER), as evidenced by subcellular localization results. This localization pattern is consistent with the majority of CYP proteins, which are known to primarily function in the ER (Neve and Ingelman-Sundberg, 2010).

Gene ontology analysis was performed to delineate gene functional classifications of orchid *CYP75*s and investigate the important biological processes they might be involved in. As a result, GO terms “response to stimulus,” “response to auxin”, “response to organic substance”, “response to endogenous stimulus”, “response to chemical”, “response to hormone”, “secondary metabolic process”, “secondary metabolite biosynthetic process”, “obsolete oxidation-reduction process”, “membrane” and “oxidoreductase activity” constituted the greatest number of genes for GO ontologies “Biological Process”, “Cellular Component,” and “Molecular Function”, respectively (**Figure S2**; **Table S7**). The data suggests that the gene ontology of orchid *CYP75*s is significantly enriched in the “Biological Process”, which is strongly linked to the production of plant metabolites. Additionally, it is heavily concentrated within response elements that relate to the plant’s reaction to external environmental factors.

### Phylogenetic analysis of orchid *CYP75s*

**Figure 2:**
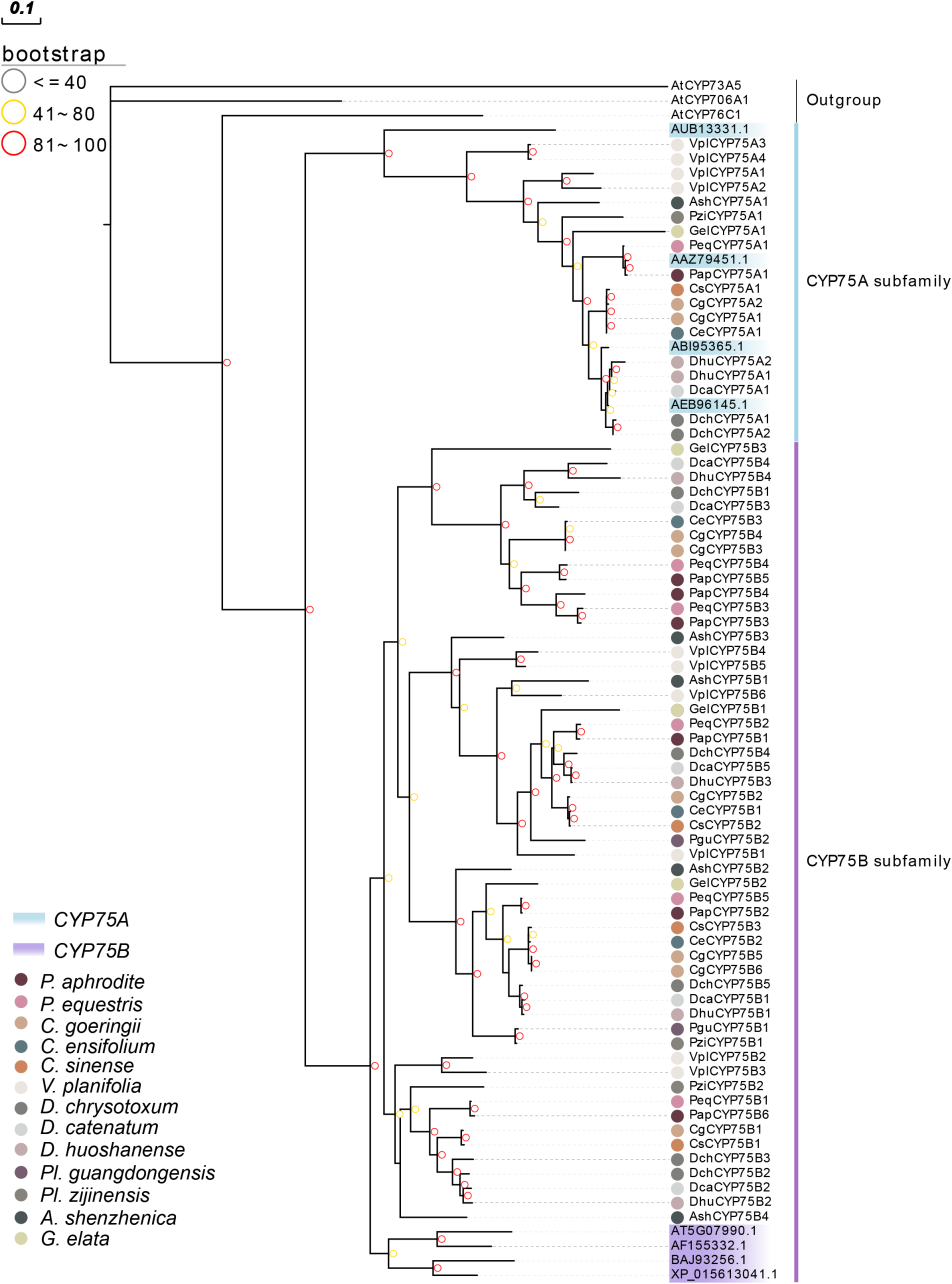
Phylogenetic tree of *CYP75* genes based on the CYP75 protein sequences of 13 orchids. The CYP75 gene family was classified into two classes: CYP75A and CYP75B. CYP75 protein sequences of all species and query sequences are available in **Table S2**.

A phylogenetic tree was constructed to analyze the evolutionary patterns of orchid *CYP75*s (**Figure 2**). 72 CYP75 proteins were used, and eight query protein sequences were used. The maximum likelihood (ML) method was used for the phylogenetic tree, which was constructed with RAxML on the CIPRES Science Gateway web server (RAxML-HPC2 on XSEDE; Miller et al., 2015). Bootstrap values were 1,000 replicates with the JTT+I+G4 model. The phylogenetic tree indicated that *CYP75*s belonged to two categories: CYP75A and CYP75B, which is consistent with the previous studies (Tanaka and Brugliera, 2013). The results clearly show that the number of members in the CYP75B subfamily has far exceeded than that of the CYP75A subfamily in the course of evolution. The branch relationships of the eight known CYP75 protein sequences are found to be correct.

### Motif variation and gene structure of orchid *CYP75*s

**Figure 3:**
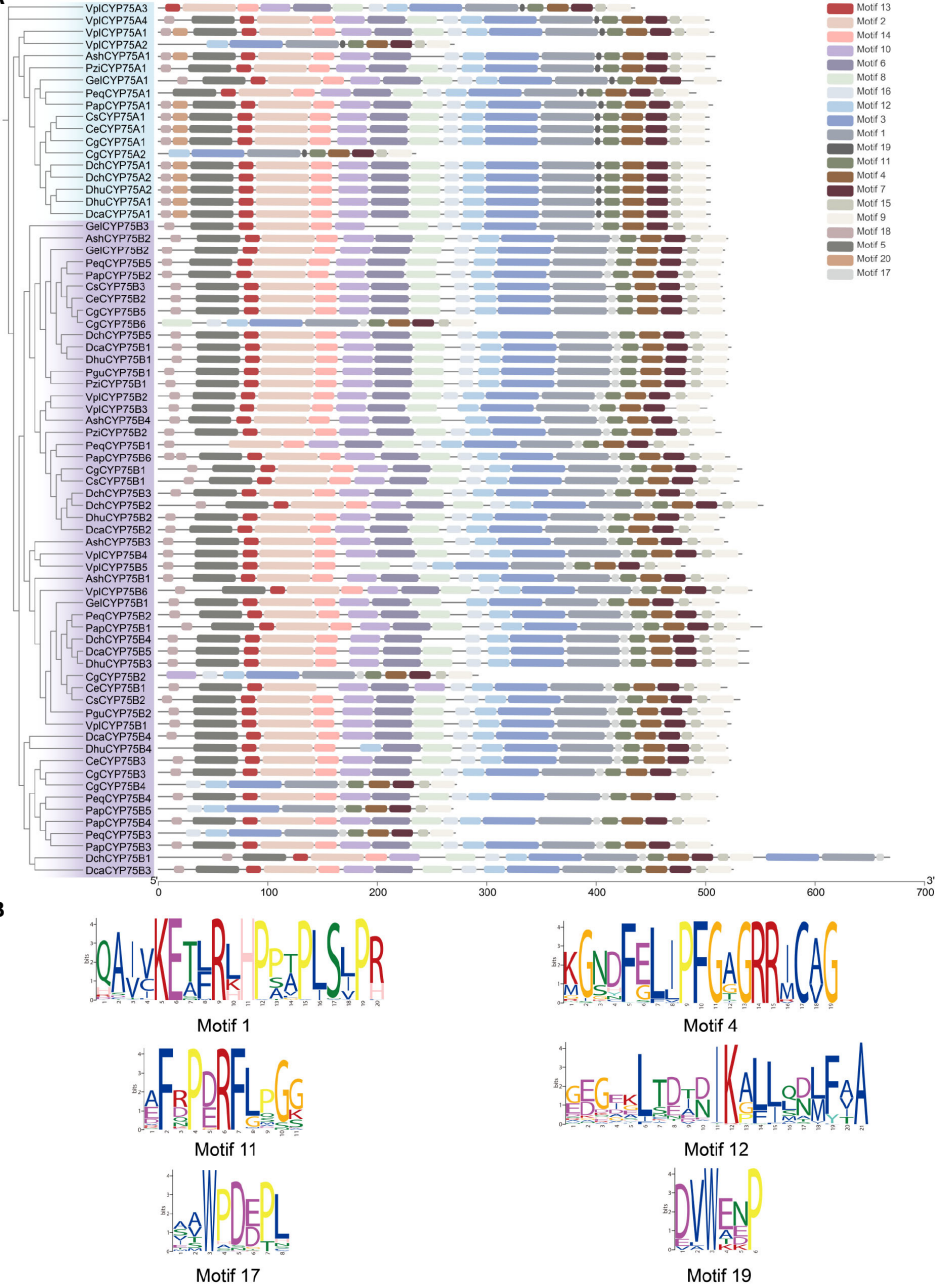
Conserved motifs of CYP75 proteins. **(A)** Predicted motifs with the phylogenetic tree of orchid *CYP75*s. **(B)** Sequence logo of motif 1, 4, 11, 12, 17, 19 which encoded the K-helix region, Heme-binding region, PERF motif, I-helix region, the *CYP75B* unique motif and the *CYP75A* unique motif, respectively.

Motifs of CYP75 proteins in 13 orchids were examined using the online analysis tool MEME, and 20 motifs were set as upper bound (**Figure 3A** **).** There are four conserved motifs commonly found in cytochrome P450 enzymes: PERF motif, K-helix region, I-helix region and the consensus sequences of the heme-binding region (**Figures 1**, **3B**), also referred to as the “P450 signature” (Crooks et al., 2004; Qi et al., 2017). As depicted in **Figure 1**, all CYP75 proteins found in orchids possess the four common CYP motifs referred to earlier. A total of 20 motifs were detected in the CYP75 proteins using the MEME software (Bailey et al., 2009). The number of CYP75 motifs ranges from nine to 18. The results show that most CYP75 proteins have identical sequence beginning with motif 18 and followed by motif 5, 13, 2, 14, 10, 6, 8, 16, 12, 3, 1, 17, 11, 4, 7, 15, ending with motif 9. Furthermore, all protein sequences of CYP75 exhibit the highly conserved motif 12, 3, 1, 17, 11, 4, 7, 15, and 9. (**Figure 3A**). Among the 20 motifs, motif 1 corresponds to K-helix region, motif 4 to heme-binding region, motif 11 to PERF motif, and motif 12 to I-helix region (**Figure 3B**). Furthermore, the unique conserved motifs in each subfamily are also found: motifs 19 are specific to the CYP75A subfamily, while motifs 17 are exclusively present in the CYP75B subfamily. This finding will greatly facilitate the identification of subfamilies within the CYP75 gene family. These 72 *CYP75*s share many common motifs, indicating high conservation. Most of the CYP75 protein sequences are conserved with differences mainly in the N terminus and intermediate regions. However, there are some CYP75 protein sequences containing a small number of motifs but also contain the typical motifs of CYP protein.

To further explore the characteristics of *CYP75* genes in orchids, intron-exon structure is analyzed as shown by **Figure S3**. The results show that the orchid CYP75 family is composed of one−eight exons and one−seven introns, and in *DchCYP75B3* and *DhuCYP75B4*, the exons are split into many small fragments by introns. Most CYP75 protein sequences have relatively long intron regions, while all *CYP75A* genes contain only one intron, which is a unique feature of the CYP75A subfamily.

### Chromosomal localization of orchid *CYP75*s

**Figure 4:**
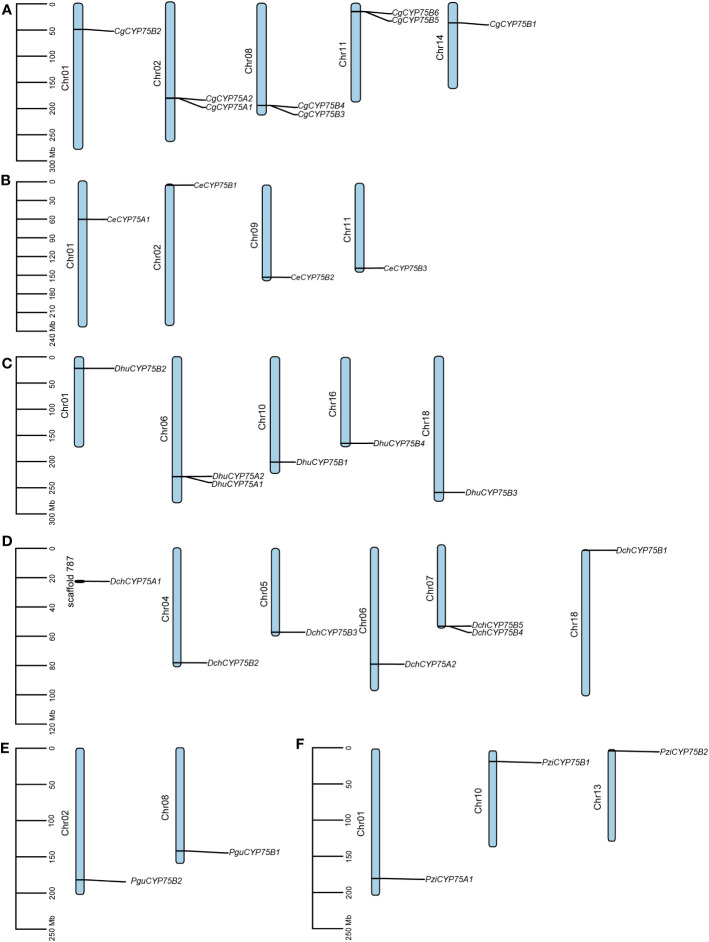
Chromosome distribution in orchids *CYP75s*. **(A)** *C. goeringii*. **(B)** *C. ensifolium*. **(C)** *D. huoshanense*. **(D)** *D. chrysotoxum*. **(E)** *Pl. guangdongensis*. **(F)** *Pl. zijinensis*.

As shown in **Figure 4A**, both *CYP75A* genes of *C. goeringii* are located on chromosome 02, while the remaining *CgCYP75B* genes are scattered on chromosomes 01, 08, 11, and 14. Among them, chromosomes 02, 08 and 11 all have two *CgCYP75*s located at the same site. The four *CYP75*s of *C. ensifolium* are scattered on chromosomes 01, 02, 09 and 11, respectively (**Figure 4B**). The *CYP75* genes of *D. huoshanense* are evenly distributed across five chromosomes, specifically chromosomes 01, 06, 10, 16, and 18. The two *DhuCYP75A* genes are located together on chromosome 06 (**Figure 4C**). In *D. chrysotoxum*, only *DchCYP75B4* and *DchCYP75B5* are co-located on chromosome 07, while the remaining *DchCYP75* are separately distributed on various chromosomes. Among them, *DchCYP75A1* has not been assembled onto the chromosome, but is located on scaffold 787 (**Figure 4D**). For both *Platanthera* species, two *PguCYP75Bs* are co-located on chromosome 02 and 08 of *Pl. guangdongensis*, while three *PziCYP75*s are individually located on chromosome 01, 10 and 13 of *Pl. zijinensis* (**Figures 4E, F**).

### Collinearity analysis of CYP75 gene family in three orchids

**Figure 5:**
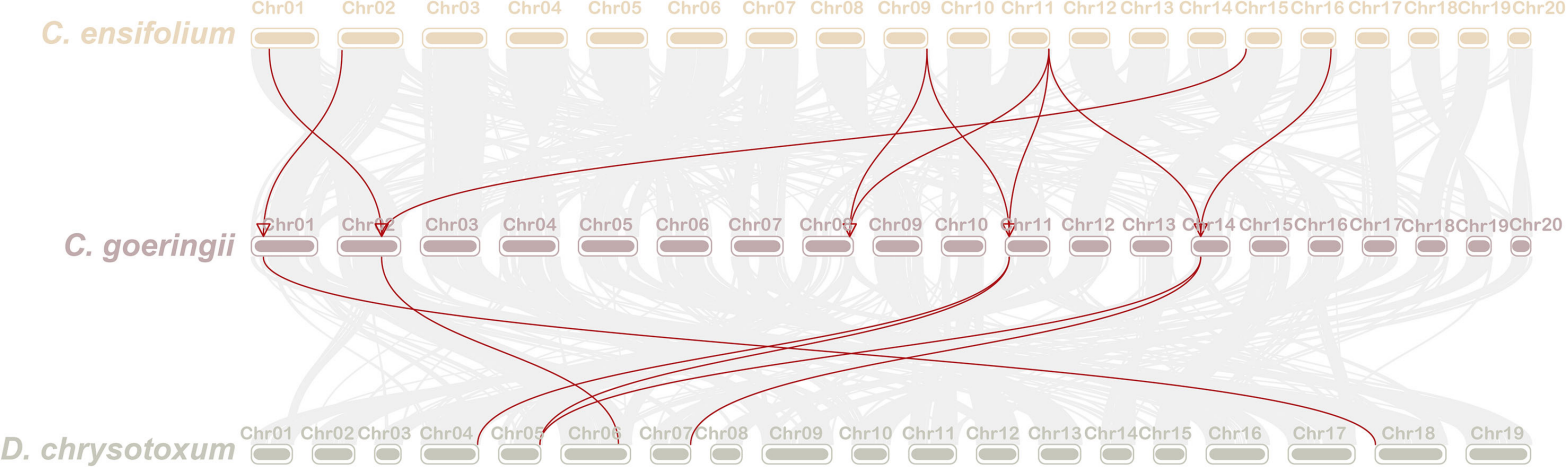
The collinearity of CYP75 genes between *C. goeringii* and *C. ensifolium*, *C. goeringii* and *D. chrysotoxum*.

To investigate the evolution of *CYP75* genes in orchids, the collinear relationship among *CYP75*s in *C. goeringii*, *C. ensifolium*, and *D. chrysotoxum* was analyzed. Our collinear analysis revealed a one-to-one correspondence among all *CYP75* genes in the three orchids, indicating limited reshuffling of *CYP75* orthologs and significant genomic rearrangements following the divergence of *Dendrobium* and *Cymbidium* lineages (**Figure 5**). Furthermore, we also examined that *CYP75* gene tandem duplication occurred on the chromosomes of both *C. goeringii* and *D. chrysotoxum*, which was consistent with the results of chromosome localization (**Figure 4**). Our results suggest that a small-scale tandem duplication may have led to the expansion of *CYP75* gene family in orchids.

### *Cis*-acting regulatory elements of *CgCYP75*s

**Figure 6:**
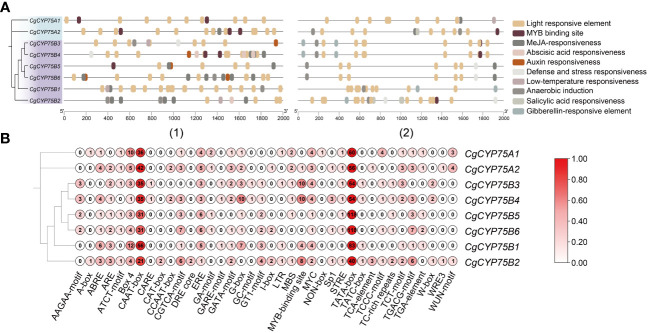
*Cis*-acting elements in the 2k bp of upstream and downstream regions of *CgCYP75* genes. **(A)** Elements with similar regulatory functions are displayed in the same color. (1) The 2k bp of upstream of *CgCYP75* genes. (2) The 2k bp of downstream of *CgCYP75* genes. **(B)** Numbers of each type of element.

To explore the regulatory roles of *CYP75*s, we retrieved the 2,000 bp upstream and downstream regions of *CYP75* genes in *C. goeringii* to identify potential *cis*-elements. We identified a total of 1,257 *cis*-acting elements, including 38 types and ten responsive functions (**Figure 6**; **Table S4**). Among these elements, TATA-box made up the most common elements (46.38%), followed by CAAT-box (22.83%) (**Table S5**). *Cis*-element functions included phytohormone responsiveness for gibberellin, auxin, methyl jasmonate (MeJA), salicylic acid, and abscisic acid (ABA); stress responses, such as anoxic, anaerobic, low-temperature, and defense; and growth and development elements, such as light response and MYB binding site (**Figure 6**). Each *CgCYP75* gene contained multiple types of elements with light responsiveness as the most occurring element function (**Figure 6**), supporting that light is one of the most important environmental factors affecting flavonoid biosynthesis in plants (Zoratti et al., 2014). The second and third most abundant types of elements identified were MeJA-responsive and ABA-responsive elements (**Table S4**). The results suggest that these elements may play a role in modulating these two phytohormones. Of particular note are the MYB binding site elements, which are key transcription factors involved in pigment synthesis. The significant proportion of these elements further emphasizes the critical role of the *CYP75* genes in the synthesis of plant pigments.

### Expression patterns of orchid *CYP75*s and qRT-PCR verification of *CgCYP75*s

**Figure 7:**
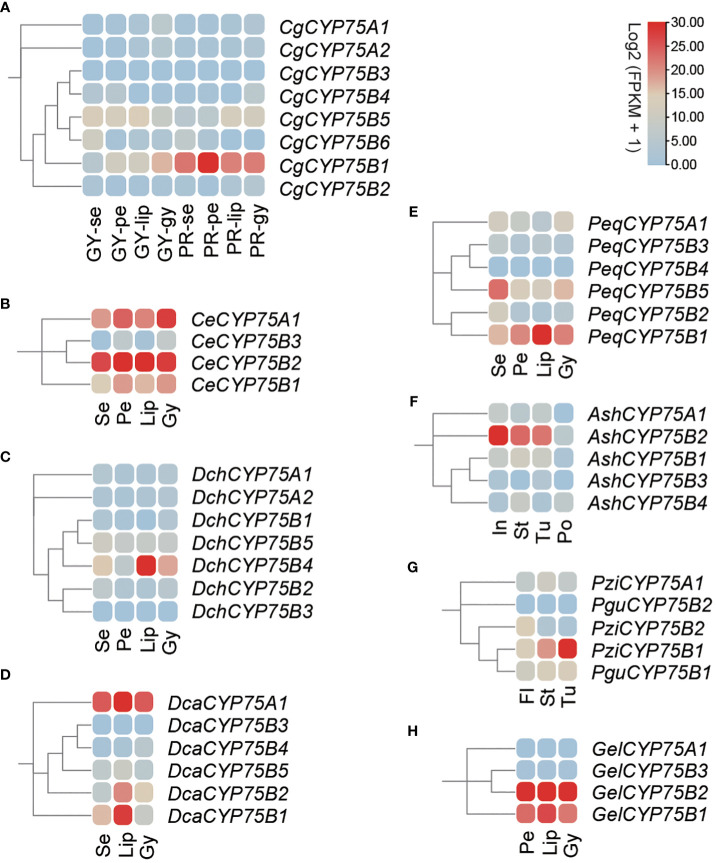
The expression patterns of *CYP75* genes among different tissues in nine orchids. Se, sepal; pe, petal; lip, labellum; gy, gynostemium; in, inflorescence; st, stem; tu, tuber; po, pollinium; fl, whole flower. **(A)** *C*. *goeringii.* GY, green-yellow flower; PR, purple-red flower. **(B)** *C*. *ensifolium*. **(C)** *D*. *chrysotoxum*. **(D)** *D*. *catenatum*. **(E)** *P. equestris.* **(F)** *A*. *shenzhenica*. **(G)** *Pl. guangdongensis* and *Pl. zijinensis*. **(H)** *G*. *elata*. The FPKM values of orchid *CYP75*s in different flower organs are listed in **Table S6**.

The orchid transcriptomic data from nine orchids out of 13 were visualized as heatmaps via TBtools software as shown in **Figure 7**. Expression analysis was conducted using transcriptome data from nine orchids, including various plant organs, such as sepals, petals, labellums (also known as lips), the gynostemium, inflorescence, stems, tubers, pollinia, and complete flowers. The expression profile showed that *CYP75B* genes were expressed broadly in flower organs, while the expression of *CYP75A* genes was more confined to *C. ensifolium*, *D. catenatum* and *G. elata*, particularly in the gynostemiums (**Figure 7**). In *C. goeringii*, *CYP75A* genes showed little expression in all tissues of two varieties, while *CgCYP75B1* exhibited an exclusive expression in four organs of ‘PR’ *C. goeringii* and gynostemium of ‘GY’ *C. goeringii* (**Figure 7A**). Furthermore, *CeCYP75B1* and *CeCYP75B2* exhibited elevated expression levels across all four floral organs (**Figure 7B**). In *D. chrysotoxum*, the expression of the *DchCYP75B4* gene was significantly higher on the lip than other parts, due to the predominant distribution of its anthocyanin in the red macula of the lip (**Figure 7C**). A similar situation existed in *D. catenatum*, *DcaCYP75B1* and *DcaCYP75B3* were highly expressed in the lip with purple-red spots (**Figure 7D**). *PeqCYP75B1* was expressed prominently in various tissues of *P. equestris*, with higher expression in the darker colored lip region. On the other hand, *PeqCYP75B5* showed significant expression levels in the sepals. (**Figure 7E**). *AshCYP75B3* of *A. shenzhenica* was expressed in all parts except for the pollinium, with the highest expression in the inflorescence (**Figure 7F**). In both of the *Platanthera* species, *PziCYP75B1* and *PguCYP75B4* exhibited the highest expression levels in fleshy underground tubers (**Figure 7G**), possibly indicating their involvement in non-biological stress response (Iwashina, 2003; Pourcel et al., 2007; Zhang et al., 2020). Similarly, *G. elata* was a mycoheterotrophy orchid without anthocyanins in its tissues, and the expression of *GelCYP75A* and *GelCYP75B* were comparable in various tissues (**Figure 7H**). Their functions are not related to anthocyanin synthesis but are possibly associated with physiological activities required for adaptation to its unique habitat (Iwashina, 2003; Pourcel et al., 2007; Zhang et al., 2020).

**Figure 8:**
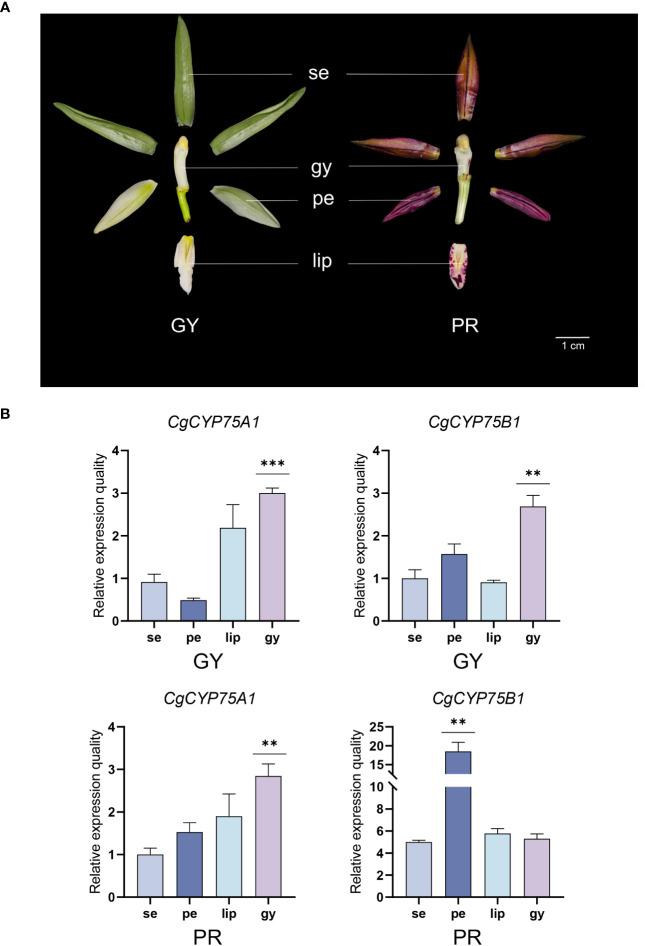
Expression profiles of different tissues of *CgCYP75* genes by real-time reverse transcription quantitative PCR (RT-qPCR). **(A)** Two flower colour types. GY, green-yellow flower; PR, purple-red flower; se, sepal; pe, petal; lip, labellum; gy, gynostemium. **(B)** RT-qPCR validation of transcriptomic data of the *CgCYP75A1* and *CgCYP75B1* at four flower organs. The error bars indicate three RT-qPCR biological replicates. The asterisk indicates the P value in the significance test (** p < 0.01, *** p < 0.001).

To further investigate the specific roles of *CYP75* gene expression in two *C. goeringii* varieties, their sepals, petals, lips and gynostemiums were analyzed by qRT-PCR (**Figure 8**). In two *C. goeringii* varieties (**Figure 8A**), the *CgCYP75B1* showed extremely high expression in ‘PR’ *C. goeringii*, especially in petal which matched the transcriptome data, but were barely detected in ‘GY’ *C. goeringii*, further verifying that *CYP75B* genes have an anthocyanin-specific expression in the flower organs. As for *CgCYP75A1*, its expression level was relatively low in four types of tissues of the two *C. goeringii* plants (**Figure 8B**). Interestingly, *CgCYP75A1* and *CgCYP75B1* showed a certain degree of stable expression in the gynostemiums of both *C. goeringii* plants (**Figure 8B**). The function of these *CYP75* genes may not be related to anthocyanin synthesis, but rather to attracting pollinators to promote reproduction (Samanta et al., 2011).

## Discussion

Flower color is one of the key criteria for evaluating the quality of ornamental plants, as well as their horticultural and economic values. Plant flower color is also a major factor in attracting pollinators, which helps to increase the success rate of pollination and plays an important role in the evolution of plants (Whibley et al., 2006; Hopkins and Rausher, 2012; Mu et al., 2017). Orchidaceae contains the most colorful plants in the world with a rich variety of colors and characteristics (Roberts and Dixon, 2008). As one of the earliest identified gene families in the CYP450 superfamily, *CYP75* plays an important role in regulating plants’ flavonoids biosynthesis and the synthesis of flower pigments (Ayabe and Akashi, 2006; Tanaka and Brugliera, 2013). How the CYP75 gene family affects the flower color formation process and a series of physiological processes in orchids by regulating flavonoid biosynthesis is an interesting topic. In this research, a total of 72 *CYP75* genes were identified from 13 orchids, according to their respective quantities, suggesting that the CYP75 gene family consists of a small number of copies (**Table 1**). In this study, the amount of *CYP75* varied significantly among each orchid, ranging from two to ten. All extant orchids share a common whole-genome duplication (WGD) event in their ancestry, which was followed by varying degrees of gene loss, resulting in the formation of five subfamilies (Zhang et al., 2017). The number of coding genes in each orchid varies substantially, with examples being 21,938 for *P. equestris* (Cai et al., 2015), 21,743 for *A. shenzhenica* (Zhang et al., 2017), 30,897 for (Chung et al., 2021; Sun et al., 2021) *C. goeringii*, and 29,044 for *V. planifolia* (Hasing et al., 2020). In addition, some orchids such as *C. goeringii*, *D. huoshanense*, and *D. chrysotoxum* have *CYP75* genes that repeat in tandem (**Figure 4**). Collinearity analyses of *CYP75* genes in *C. goeringii* and *C. ensifolium*, *C. goeringii* and *D. chrysotoxum* have also approved this (**Figure 5**). Furthermore, there were differences in genome assembly quality among the 13 orchids evaluated. Analyses of Benchmarking Universal Single Copy Orthologs (BUSCO) (Simo et al., 2015) revealed a generally low level of genome assembly completeness for orchids (**Table S1**). Consequently, the number of CYP75 genes in orchids varies widely.

Phylogenetic relationships and protein structure analyses support the division of the CYP75 gene family into two branches, CYP75A and CYP75B (**Figure 2**) which is in agreement with previous studies (Tanaka and Brugliera, 2013). Notably, no *CYP75A* gene was found in *Pl. guangdongensis*, which is similar to *A. thaliana* lacking *CYP75A* (Schoenbohm et al., 2000). The main function of the *CYP75A* genes is to regulate the precursor of blue anthocyanins (Rausher, 2006). Many blue anthocyanins pigments are formed by the presence of the trioxide B-ring of delphinium derivatives (Ayabe and Akashi, 2006). Rausher (2008) documented the macroscopic trend of flower color evolution in angiosperms, finding that flowers evolving from blue to red were more common than in the opposite situation. An illustration of this phenomenon is observed in the grape genus, whereby the peels, which is blue-violet in color, contains a significant concentration of delphinidins. This leads to an increased quantity of the *CYP75A* genes (Falginella et al., 2010). However, as for Orchidaceae, the red cyanidin color trait is more common than blue delphinidin color trait, this also explains why the *CYP75A* gene regulating F3’5’H is significantly less than the *CYP75B* gene regulating F3’H. This may be attributed to the fact that blue flower species are less abundant than red flower species in Orchidaceae. It may suggest that red hue is a favored trait in the evolution of angiosperms and the CYP75B subfamily, which governs the synthesis of anthocyanin red precursors, holds an evolutionary edge over the CYP75A subfamily. Researchers have found that some *CYP75B* genes can function as *CYP75A* genes, such as *O. sativa* and some Asteraceae plants (Lam et al., 2015). Phylogenetic analysis of existing sequences of *CYP75*s revealed that *CYP75A* was derived from *CYP75B* prior to the divergence of angiosperms and gymnosperms (Seitz et al., 2006). It is demonstrated that the *CYP75B* subfamily has perhaps a more superior evolutionary position in the CYP75 gene family, performing not only in its own function, but sometimes work as compensation for the CYP75A subfamily (Xiao et al., 2021). In this study, all orchid *CYP75*s were found to be located on the endoplasmic reticulum, as previous research has shown that CYP450 is a membrane protein primarily found on the ER membrane in eukaryotes (Brignac-Huber et al., 2016). It can be deduced that orchid *CYP75* genes are likely involved in plant metabolism and response to biotic stress via the membrane system.

All members of the CYP gene family have four identifiable motifs: the PERF motif, K-helix region, I-helix region, and heme-binding region, as shown in **Figure 1** (Crooks et al., 2004; Qi et al., 2017). In addition, this study finds specific motifs within two respective subfamilies: motif 19 is exclusive to CYP75A subfamily, while motif 17 exists only in CYP75B subfamily (**Figure 3**). These results provide more possibilities for accurate recognition of the two subfamilies in CYP75.

Whole-genome sequencing has enabled researchers to uncover variations in the gene structure of gene families across different species. While gene structure tends to be conserved within the same clade, peculiarities have been observed in the CYP75 gene family of orchids, particularly in subfamilies CYP75A and CYP75B. In contrast to the longer introns typically found in orchids, *CYP75As* has a single intron (**Figure S3**), which is a unique characteristic. Longer introns are believed to be favored during gene evolution as they increase recombination between adjacent exons, thus promoting natural selection efficiency (Jo and Choi, 2015). This unique feature of Orchidaceae may explain the extraordinary diversity of orchids.

Gene expression is primarily regulated by *cis*-acting elements of the transcription start site (Hernandez-Garcia and Finer, 2014). This study identified various types of regulatory elements within the upstream and downstream region of *CYP75* genes in *C. goeringii*, which were further classified into plant hormone response elements, stress response elements, and growth and development elements (**Figure 6**). Among these elements, the number of light response elements was the largest, indicating that light is one of the most important environmental factors influencing flavonoid biosynthesis (Zoratti et al., 2014), which is closely related to anthocyanin synthesis. Additionally, there were also a considerable number of MYB binding sites within the *cis*-acting elements of orchid *CYP75*s. MYB is one of the most important transcription factors regulating plant pigments, and it can enhance B-ring hydroxylation by upregulating *F3’5’H1* (Ma et al., 2021). Further research is needed to understand the interaction patterns between the two.

Studies have shown that the expression level of the *CYP75* genes is positively related to the accumulation of anthocyanins. Based on the RT-qPCR and transcriptome expression analysis in this study, this point of view can be supported. In two *C. goeringii* varieties with significantly different colors, the expression of the *CYP75B* genes which regulate the purple-red anthocyanins is generally much higher in ‘PR’ *C. goeringii* compared to ‘YG’ *C. goeringii*. Moreover, the expression level of *CgCYP75B* is higher than that of *CgCYP75A* in any of the two varieties. Furthermore, in the lip of ‘YG’ *C. goeringii* without anthocyanin, the *CYP75* gene shows relatively stable expression. This suggests that the function of the *CYP75* genes is not mainly to regulate anthocyanin synthesis. The two F3’H and F3’5’H enzymes regulated by the *CYP75* genes are both flavonoids, and flavonoids are involved in almost all metabolic processes in plants, including attracting pollinators (Wen et al., 2020). Orchidaceae-specific *CYP75*s probably reflect the adaptive value of efficient attraction of pollinators. These results further refine the theoretical framework of the CYP75 gene family’s relevance to flower color in orchids and provide new insights into the mechanism of color formation. Furthermore, it is now possible to alter plant color through gene silencing or introducing exogenous genes, which may solve the problem of scarce blue flowers and lay the foundation for the breeding and improvement of novel flower colors in orchids.

## Conclusion

In this study, we identified 72 members of the CYP75 gene family from the 13 orchid genomes. We analyzed their protein physicochemical properties, subcellular localization, motifs, intron-exon structures, chromosome distribution, promoter elements, expression patterns and gene ontology classification. We found that the CYP75 gene family in Orchidaceae is a low-copy gene family with high conservation. *V. planifolia* has the highest count of *CYP75* genes, with ten, while *Pl. guangdongensis* has only two genes, the *CYP75A* gene is missing and only two *CYP75B* genes are present. We observed a notable discrepancy between the number of genes in the CYP75A and CYP75B subfamilies, which could potentially be attributed to differences in their evolutionary statuses. The characteristic motifs of CYP450 are present in all CYP75 proteins, with a specific motif found in two subfamilies, respectively. We demonstrate that the distinct roles of *cis*-elements in light response and MYB binding sites are working together with the crucial function of *CYP75*s in the biosynthesis of anthocyanins. In addition, the expression patterns generated by transcriptomic and RT-qPCR data supported a color-specific expression of *CYP75B*s in the flower organs. Our study presents a comprehensive analysis of the functions and expression patterns of *CYP75* genes in Orchidaceae. These results build a foundation for deeper understanding regarding the role of *CYP75* genes in plant anthocyanin biosynthesis of plants, offering insights into the flexibility of plant pigmentation. A crucial subsequent task will entail conducting functional analysis of *CYP75* in non-model plants, in order to discern the further functions of *CYP75* in the context of angiosperms evolution.

## Data availability statement

The datasets presented in this study can be found in online repositories. The names of the repository/repositories and accession number(s) can be found in the article/**Supplementary Material**.

## Author contributions

Z-JL and SL conceived and designed the research. XZ and M-MZ prepared the original draft. XH and YH performed the data analysis. YL and SA wrote and edited the language. All authors contributed to the article and approved the submitted version.
