# Supplementary figures and images for: Genome-based identification of the CYP75 gene family in Orchidaceae and its expression patterns in Cymbidium goeringii
Source: Front Plant Sci. 2023 Sep 27;14:1243828. doi: 10.3389/fpls.2023.1243828 (PMC10564990; doi:10.3389/fpls.2023.1243828)

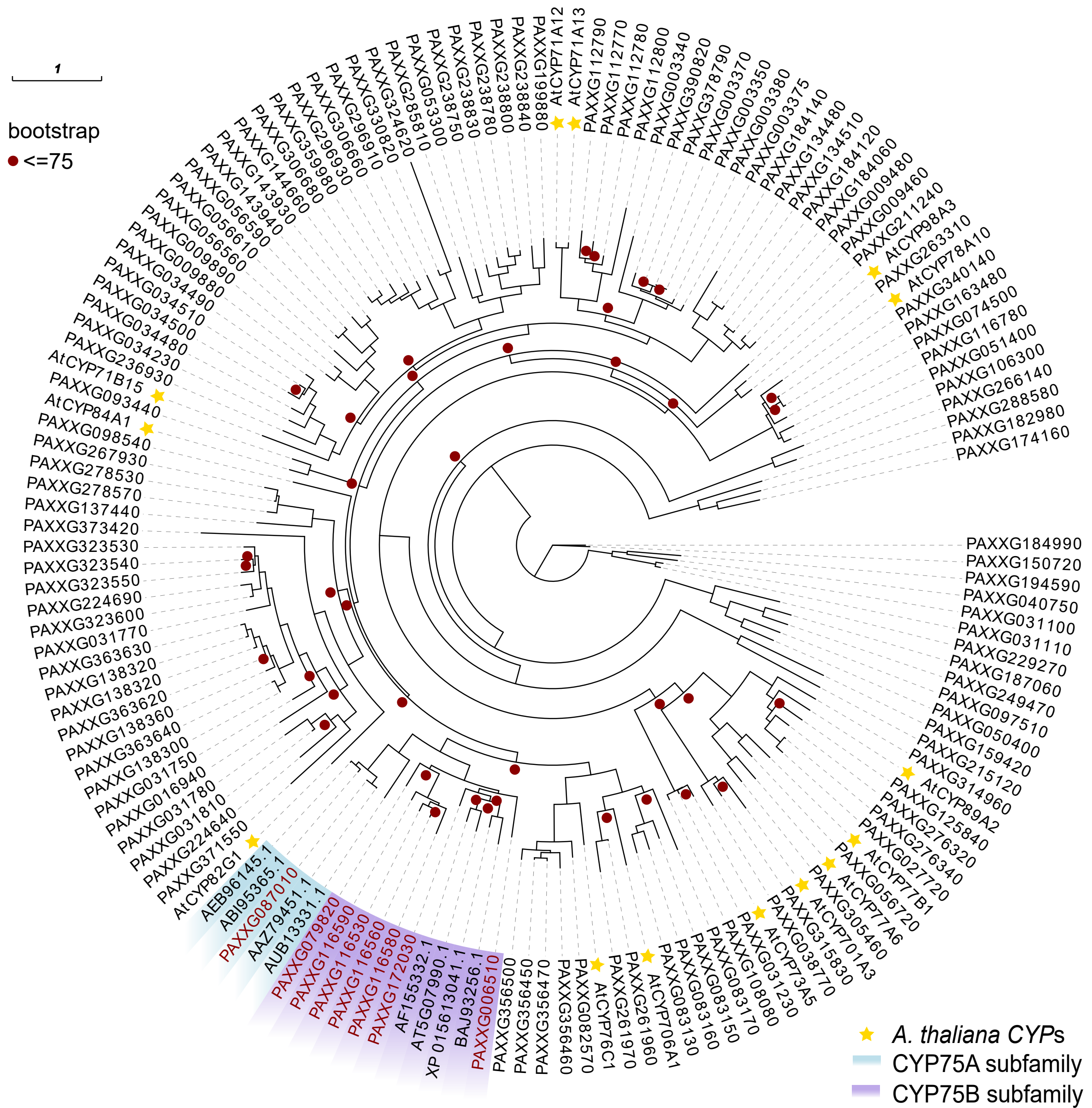

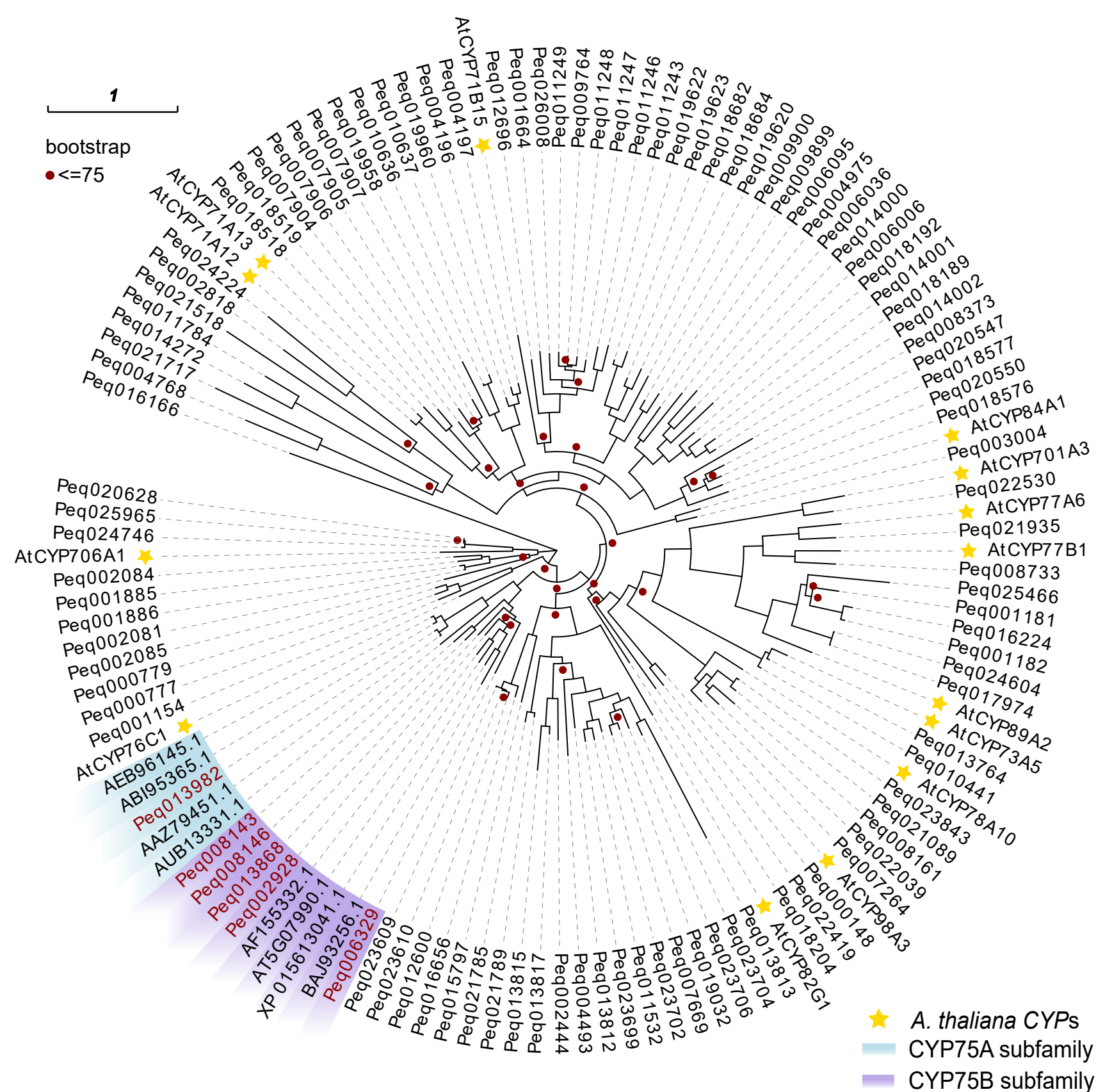

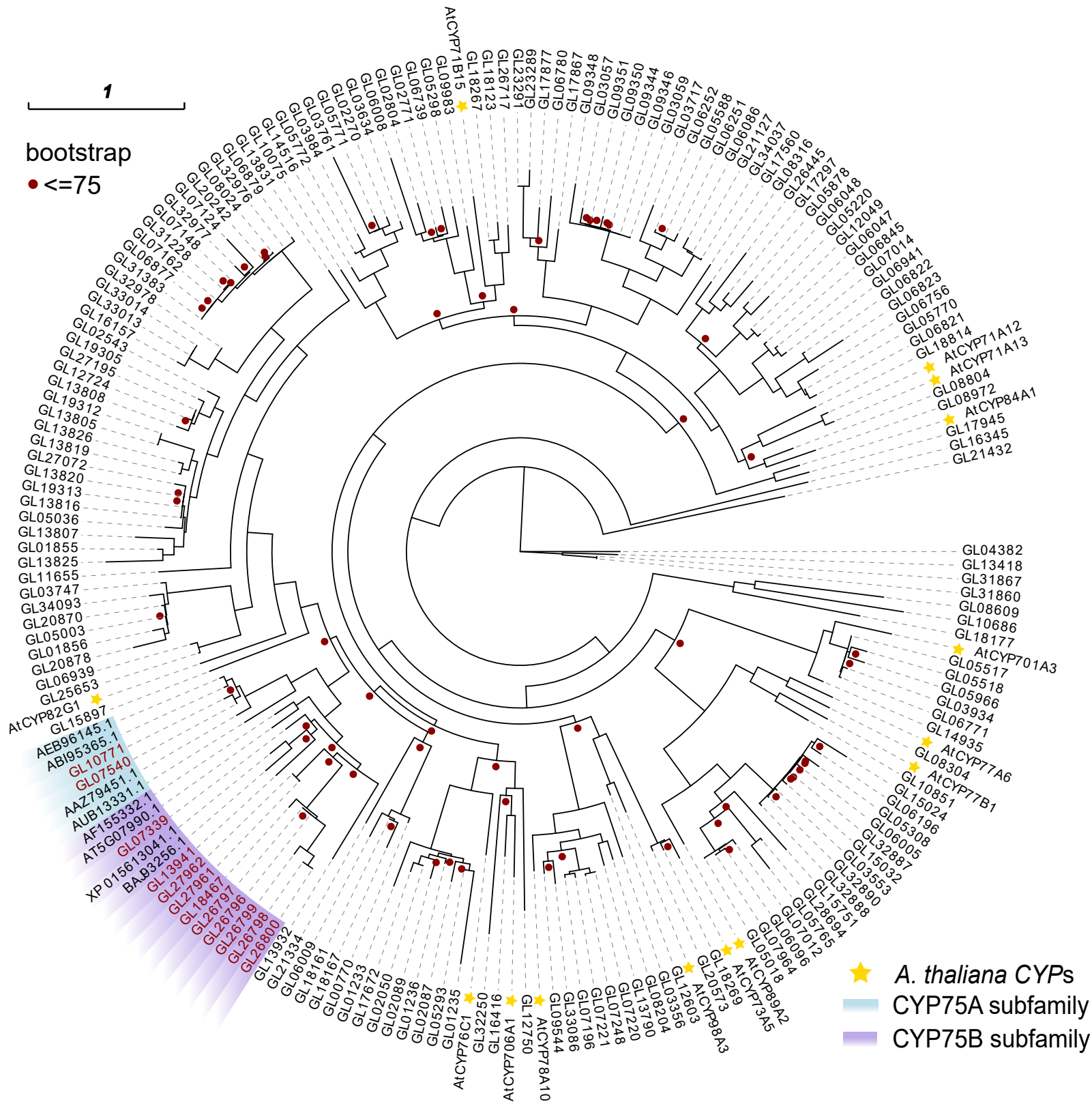

1

bootstrap

● ≤75

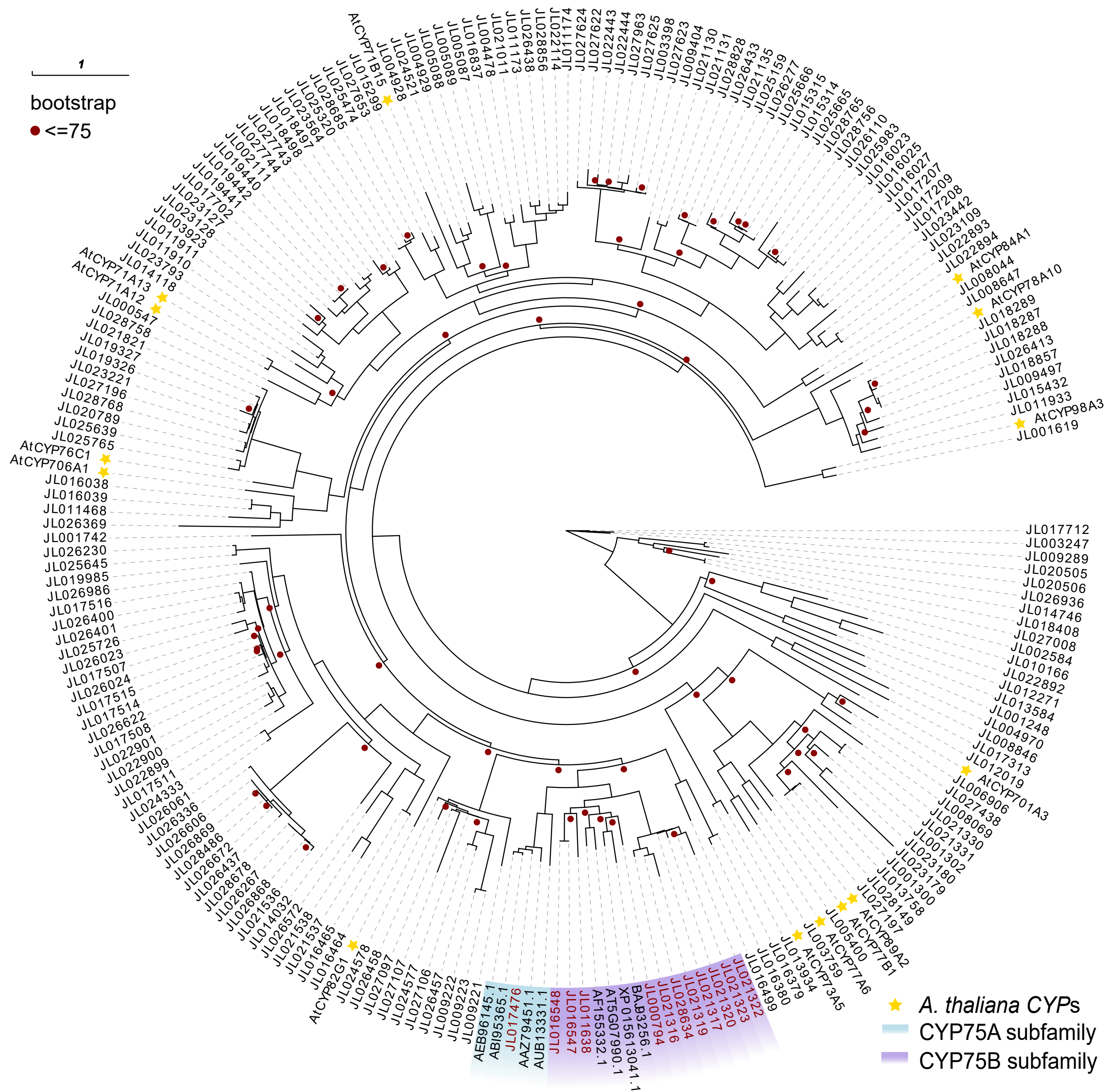

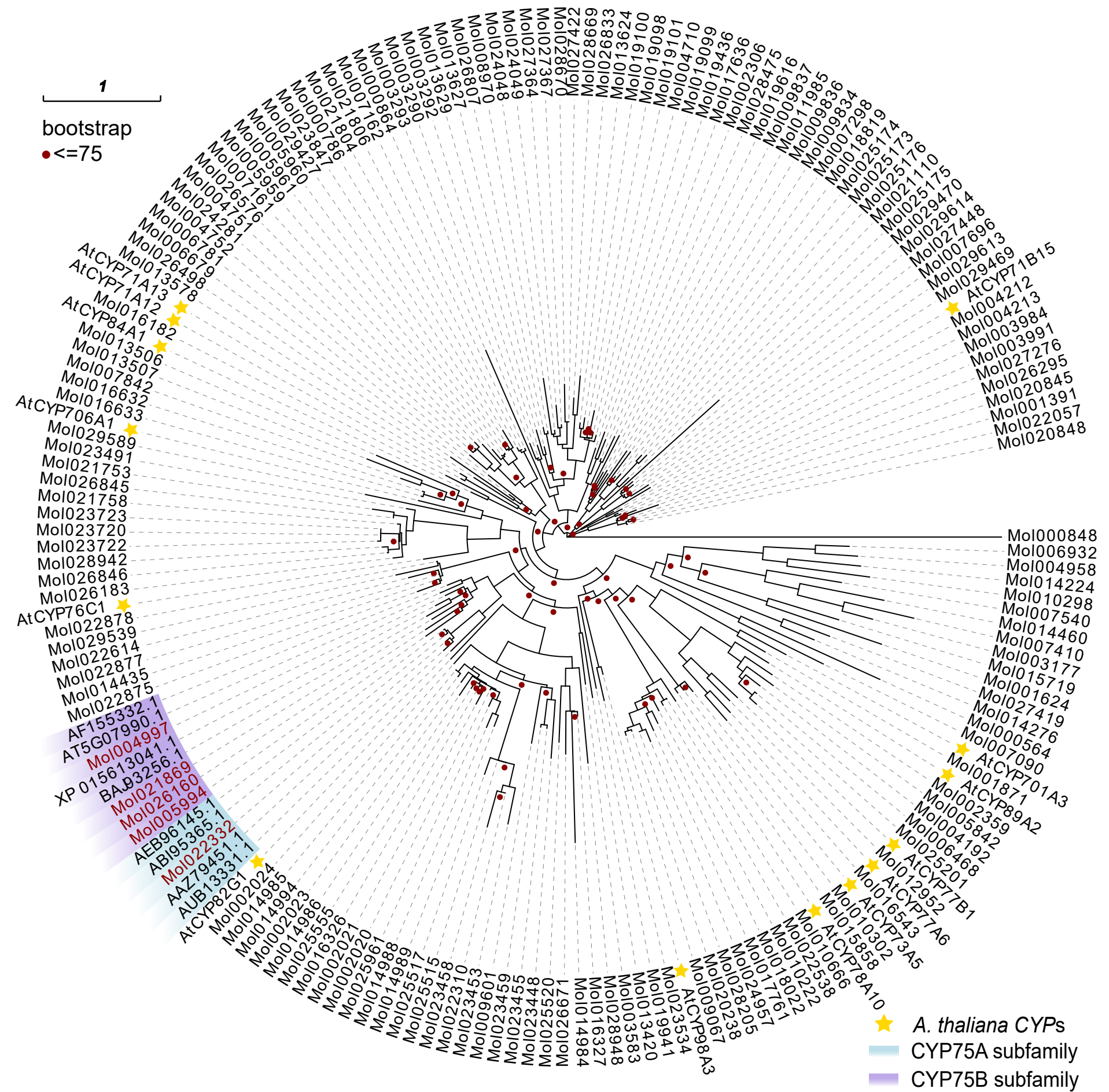

- $\leq 75$

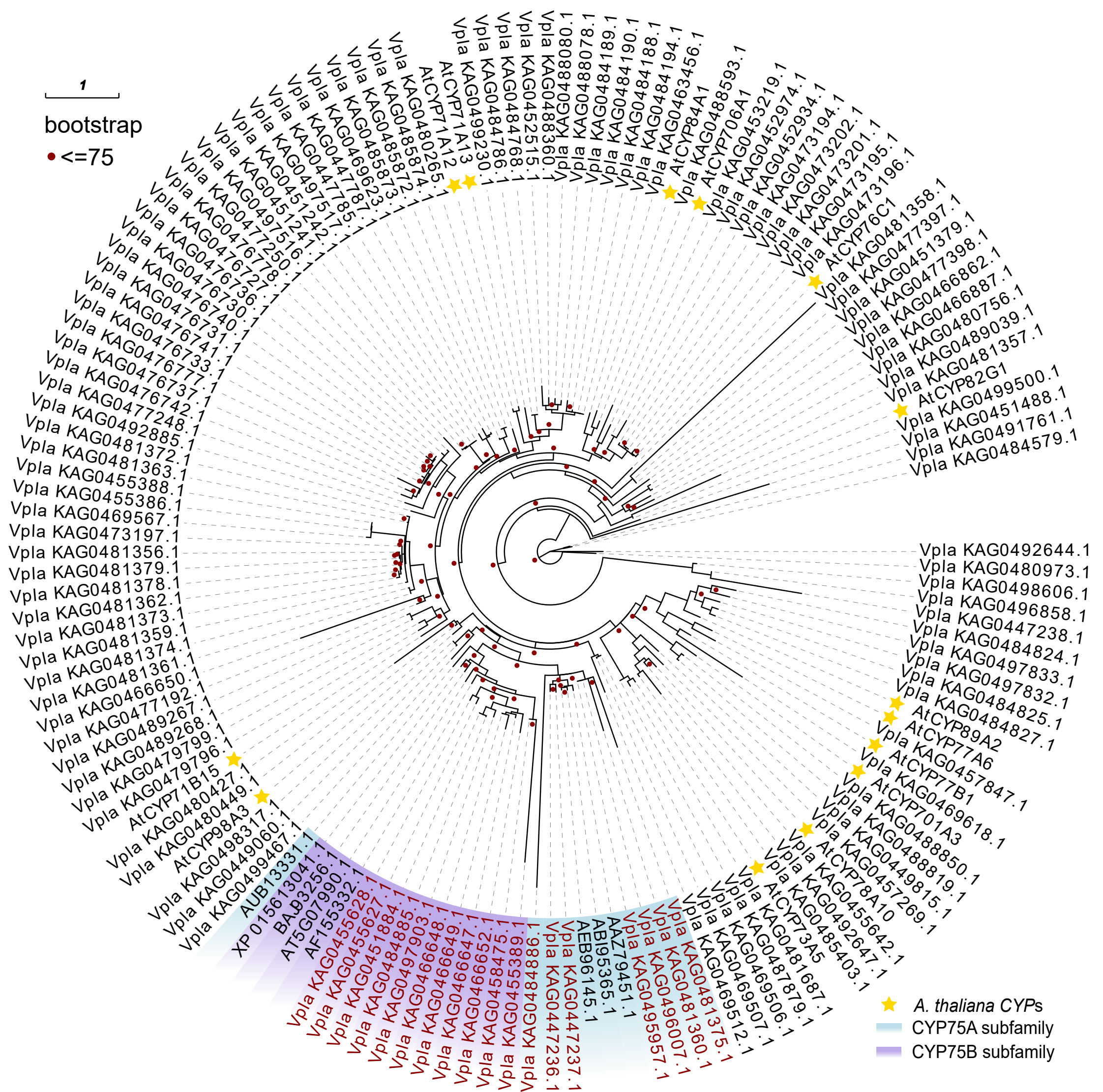

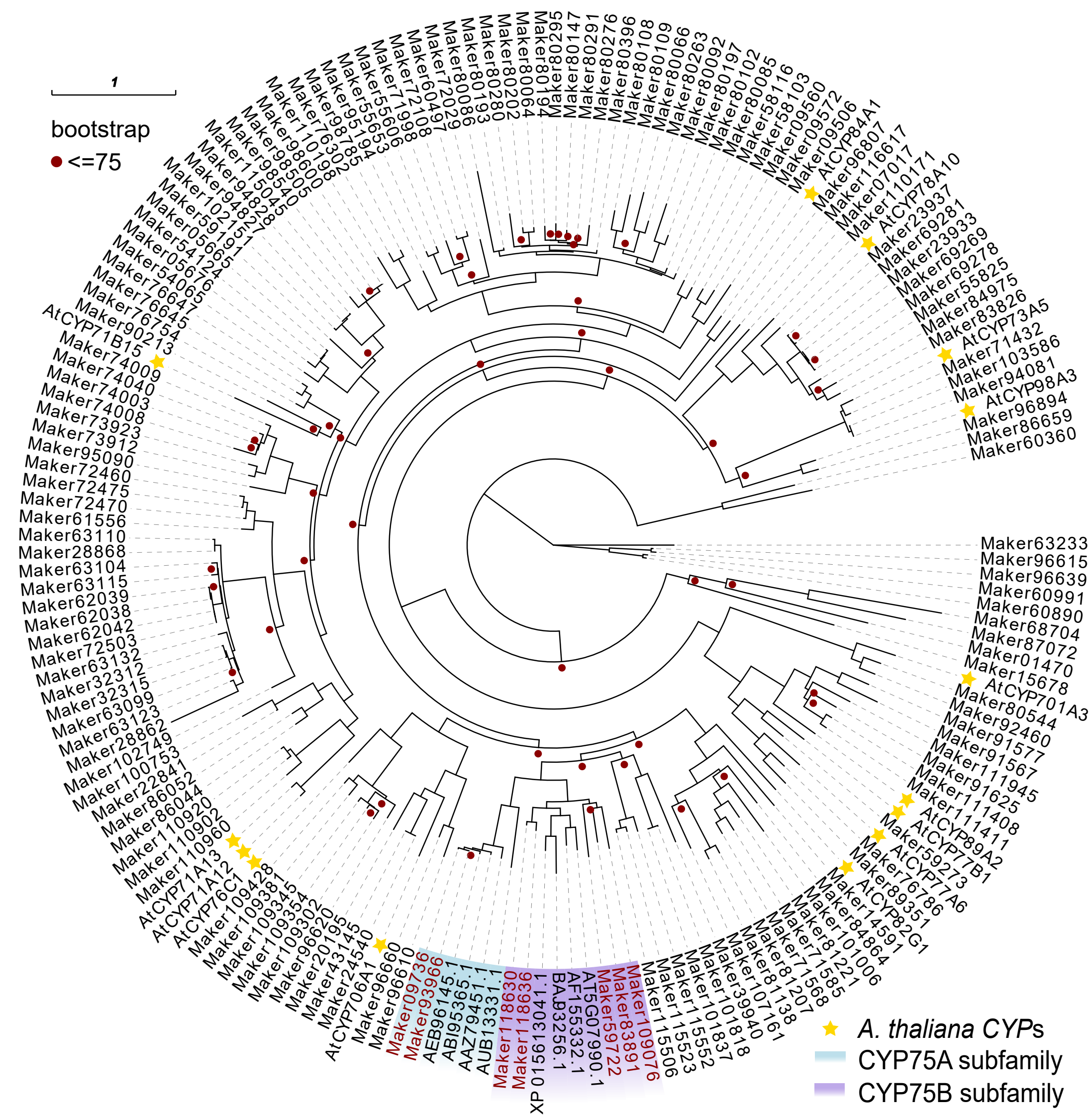

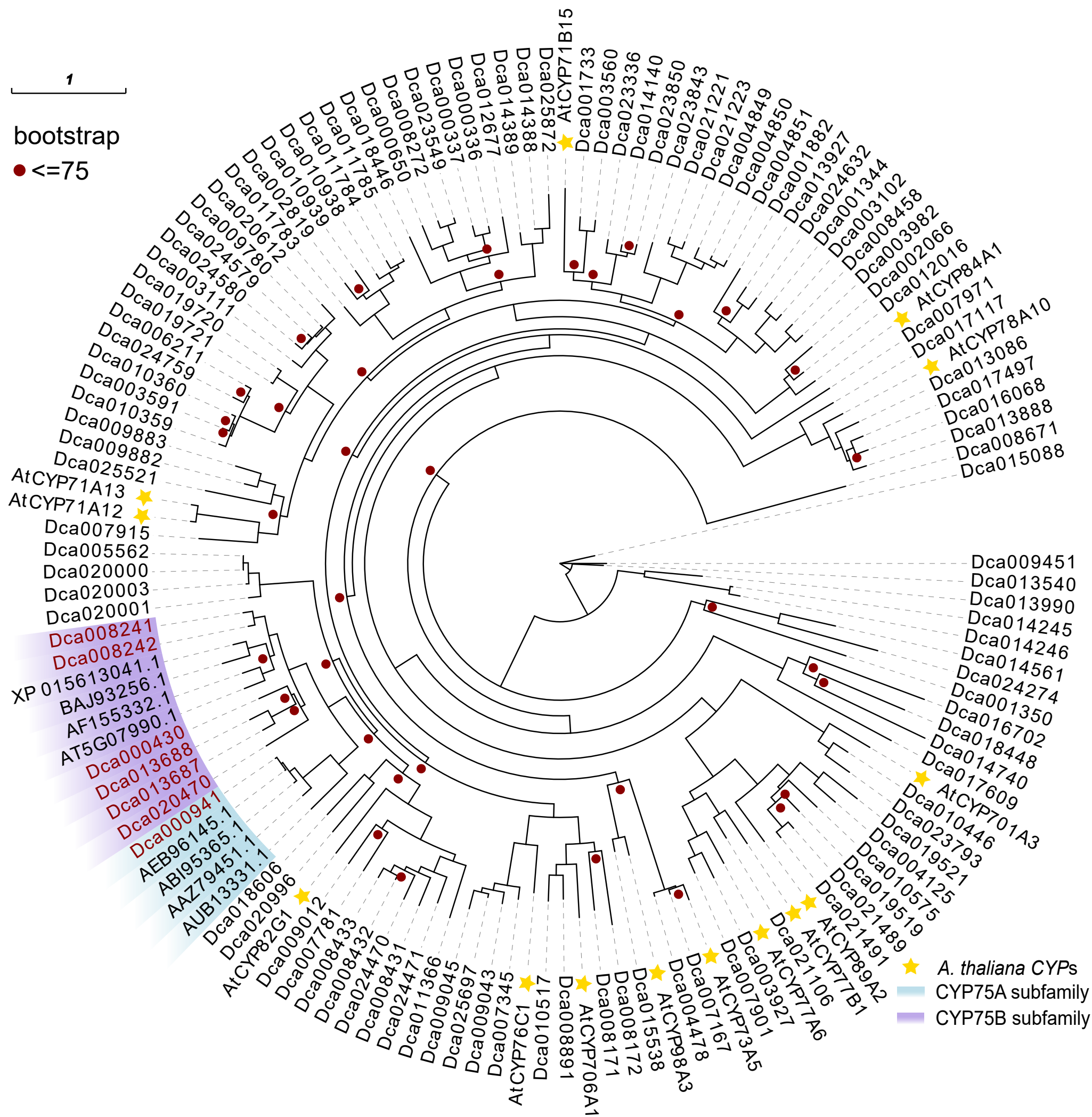

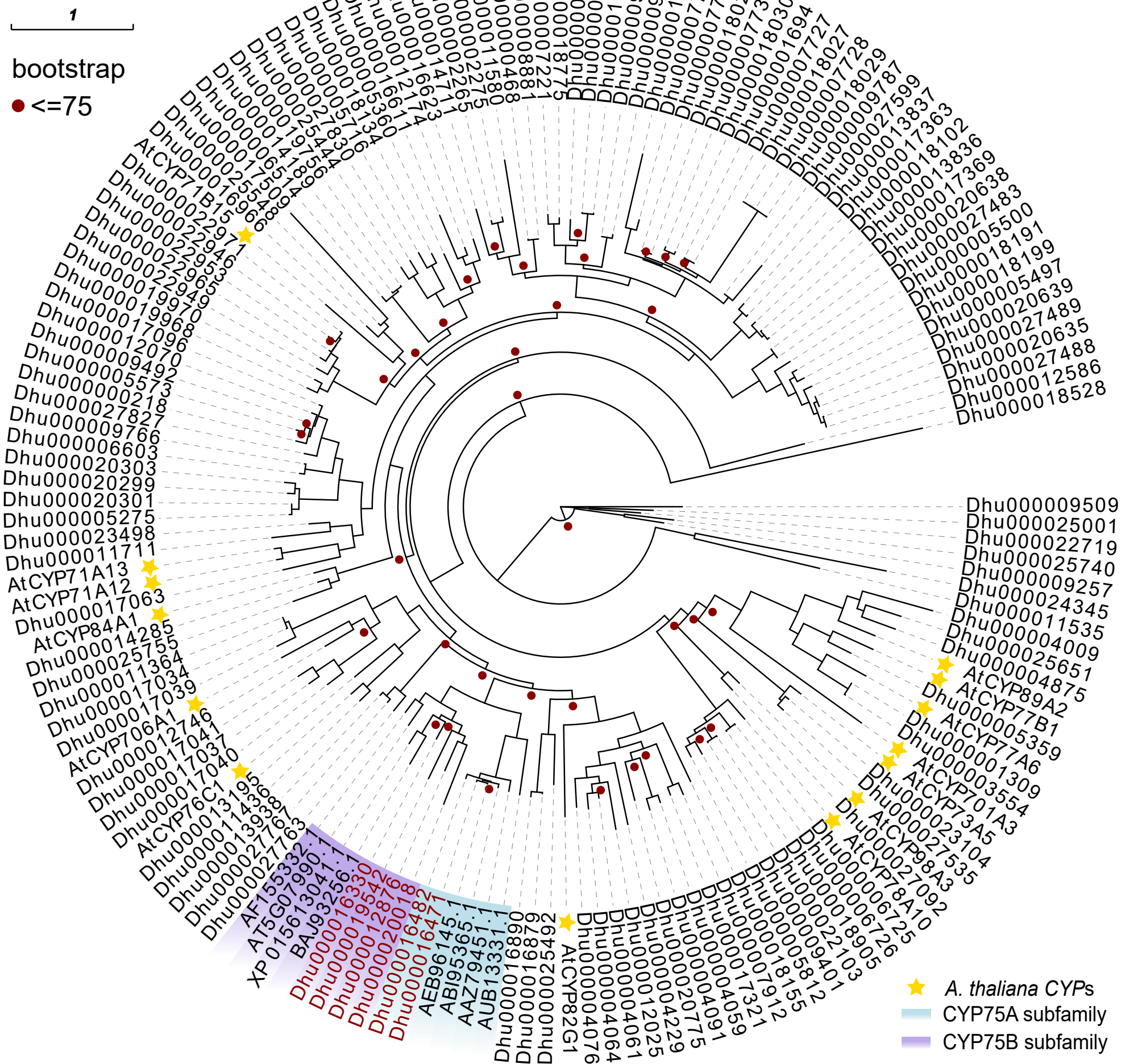

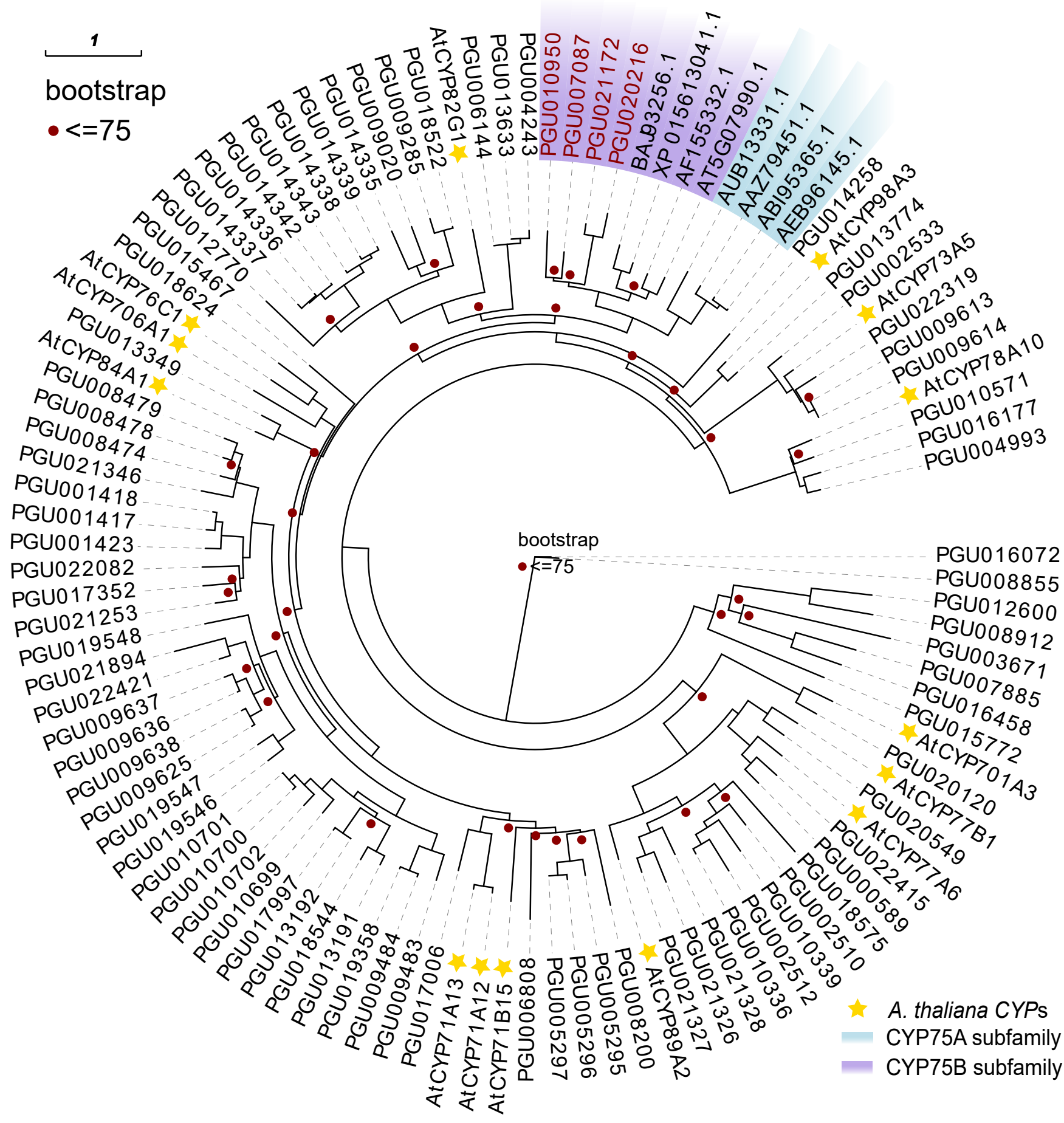

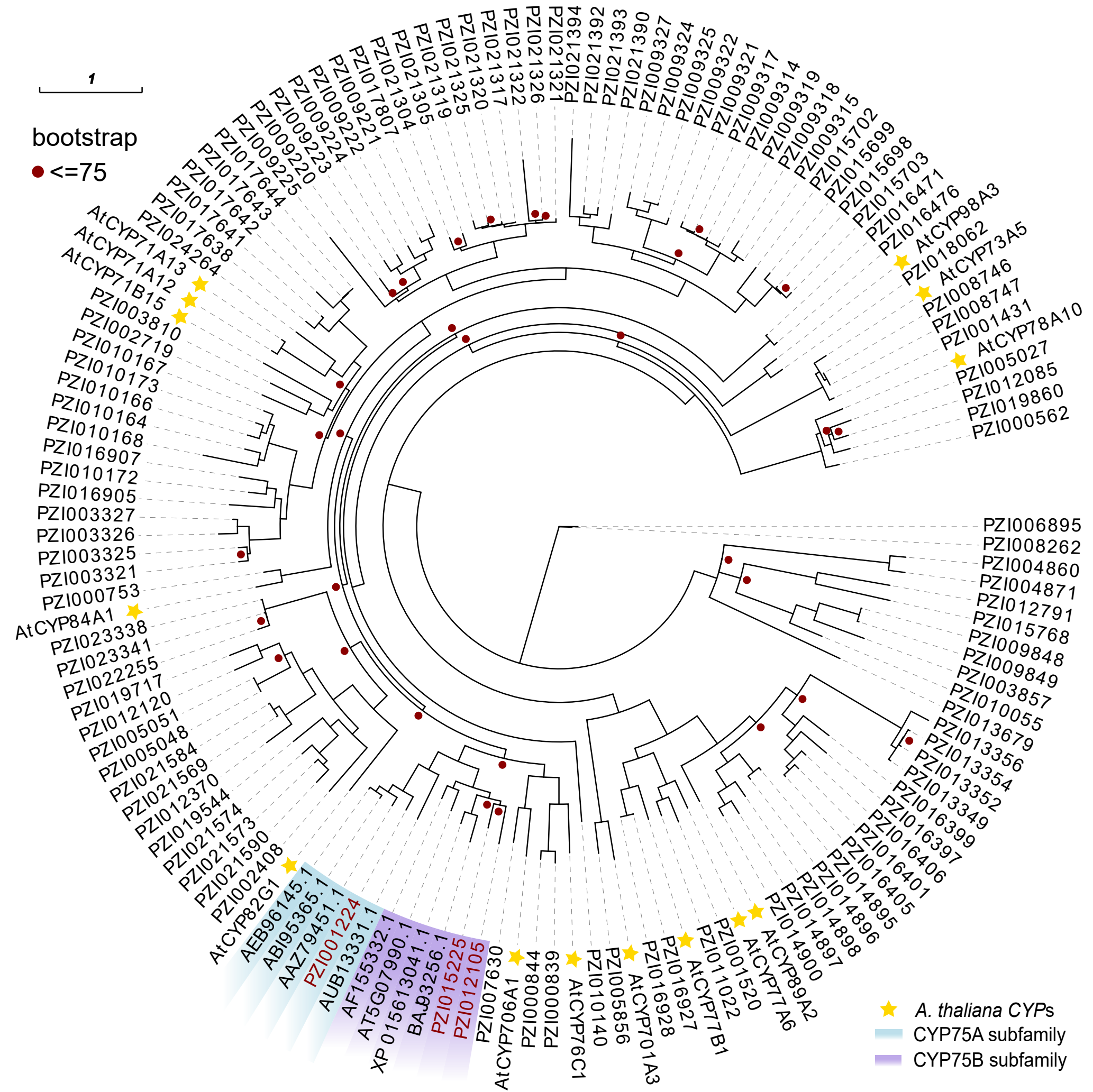



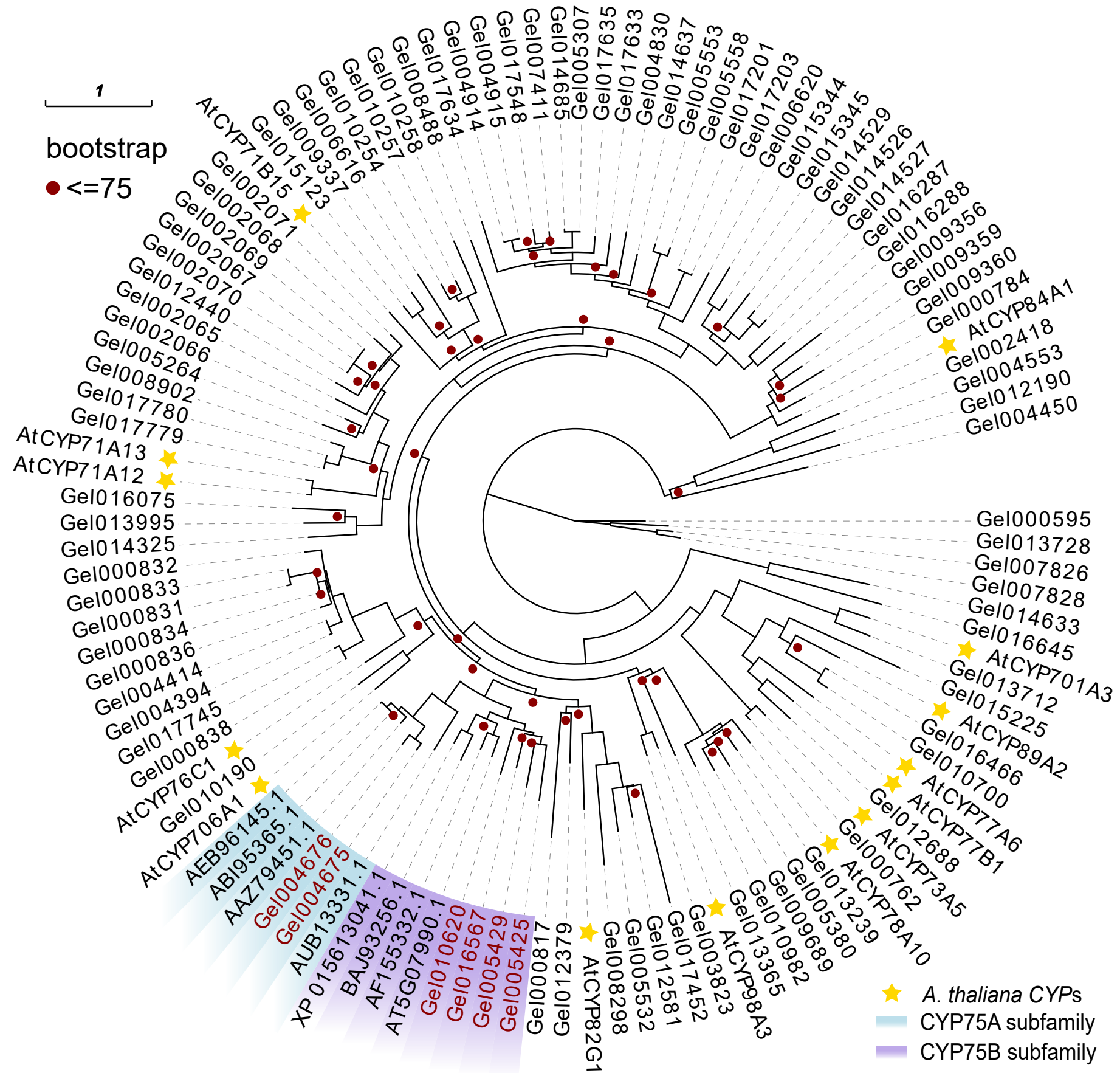

Supplement: Supplementary file 1 [file Image_1.pdf]
