# Supplementary material for: Genome-based identification of the CYP75 gene family in Orchidaceae and its expression patterns in Cymbidium goeringii
Source: Front Plant Sci. 2023 Sep 27;14:1243828. doi: 10.3389/fpls.2023.1243828 (PMC10564990; doi:10.3389/fpls.2023.1243828)
Supplement: Supplementary file 2 [file DataSheet_1.docx]

**Supplementary Information**

**Genome-based identification of the CYP75 gene family in Orchidaceae and its expression patterns in *Cymbidium goeringii***

Yuanyuan Li^1^, Xuewei Zhao^2^, Meng-Meng Zhang^2^, Xin He^2^, Ye Huang^1^, Sagheer Ahmad^1^, Zhong-Jian Liu ^1,2*^ and Siren Lan ^1,2*^

**Table S1. Download URLs for genome assemblies, annotation files, RNASeq datasets, and BUSCO evaluations for 13 orchids.**

| **Species** | **Genome assemblies and annotation** | **RNASeq datasets** | **BUSCO evaluation** |
| --- | --- | --- | --- |
| *P. aphrodite* | The National Center for Biotechnology Information (NCBI) BioProject database (https://www.ncbi.nlm.nih.gov/bioproject/; accession number: PRJNA383284). |  | 95.0% (Chao et al., 2018) |
| *P. equestris* | NCBI BioProject database (https://www.ncbi.nlm.nih.gov/bioproject/; accession number: PRJNA192198). | OrchidBase (http://orchidbase.itps.ncku.edu.tw/). | 94.0% (Cai et al., 2015) |
| *C. goeringii* | NCBI BioProject database (https://www.ncbi.nlm.nih.gov/bioproject/; accession number: PRJNA749652)*.* | BioProject database (https://ngdc.cncb.ac.cn/bioproject/; accession number: PRJCA009885). | 86.9% (Sun et al., 2021) |
| *C. ensifolium* | The Biological Project Library (BioProject) database (https://ngdc.cncb.ac.cn/bioproject/; accession number: PRJCA005355). | BioProject database (https://ngdc.cncb.ac.cn/bioproject/; accession number: PRJCA005426). | 87.0% (Ai et al., 2021) |
| *C. sinense* | National Genomics Data Center BioProject database (https://ngdc.cncb.ac.cn/; accession number: PRJCA005619). |  | 91.0% (Yang et al., 2021) |
| *V. planifolia* | NCBI BioProject database (https://www.ncbi.nlm.nih.gov/bioproject/; accession number: PRJNA633886). |  | 86.0% (Hasing et al., 2020) |
| *D. chrysotoxum* | NCBI BioProject database (https://www.ncbi.nlm.nih.gov/bioproject/; accession number: PRJNA664445). | | 90.3% (Zhang et al., 2021) |
| *D. catenatum* | NCBI BioProject database (https://www.ncbi.nlm.nih.gov/bioproject/; accession number: JSDN00000000). | BioProject database (https://ngdc.cncb.ac.cn/bioproject/; accession number: PRJNA783177). | 93.0% (Zhang et al., 2016) |
| *D. huoshanense* | NCBI BioProject database (https://www.ncbi.nlm.nih.gov/bioproject/; accession number: PRJNA597621). |  | 86.6% (Han et al., 2020) |
| *Pl. guangdongensis* | NCBI BioProject database (https://www.ncbi.nlm.nih.gov/bioproject/; accession number: PRJNA739531). | | 72.06% (Li et al., 2022) |
| *Pl. zijinensis* | NCBI BioProject database (https://www.ncbi.nlm.nih.gov/bioproject/; accession number: PRJNA739531). | | 88.66% (Li et al., 2022) |
| *A. shenzhenica* | NCBI BioProject database (https://www.ncbi.nlm.nih.gov/bioproject/; accession number: PRJNA310678). | | 93.62% (Zhang et al., 2017) |
| *G. elata* | BioProject database (https://ngdc.cncb.ac.cn/; accession number: PRJCA005619). | | 90.0% (Xu et al., 2021) |

**Table S2. CYP75 protein sequences of 13 orchids and query sequences (see separate file).**

**Table S3. The primers of *CYP75* in *C. goeringii* and their internal reference genes.**

| **Genes** | **(5’–3’) Forward primer** | **(5’–3’) Reverse primer** |
| --- | --- | --- |
| *CgActin* (internal reference) | ATTCAGCCTCTAGTTTGCGATAA | CAGCAAATCCAGCCTAACAAATG |
| *CgCYP75A1* | CCATCTCCTCGGTTCGAAGG | ACATTCGCCATGGCACAAAC |
| *CgCYP75B1* | GCCACTATCCTCTTCGCCTC | AGCGTGTAGGGTTTGGTGAG |

**Table S4. *Cis*-acting elements in the 2k bp of upstream and downstream regions of *CgCYP75*s** **(see separate file).**

**Table S5. *Cis*-acting element types and numbers of *CgCYP75*s (see separate file).**

**Table S6. The FPKM values of orchid *CYP75*s in different flower organs (see separate file).**

**Table S7. Gene ontology (GO) annotation of orchid *CYP75*s (see separate file).**

**Figure S1. The phylogenetic tree of *CYP*s for each orchid (see separate file).**

**
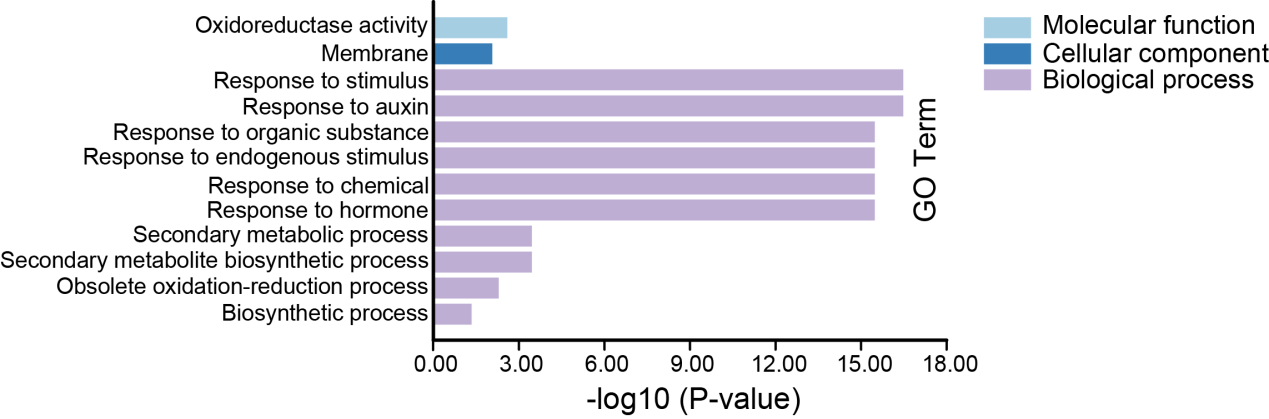
**

**Figure S2. Gene ontology (GO) classification of *CYP75* genes.**

**
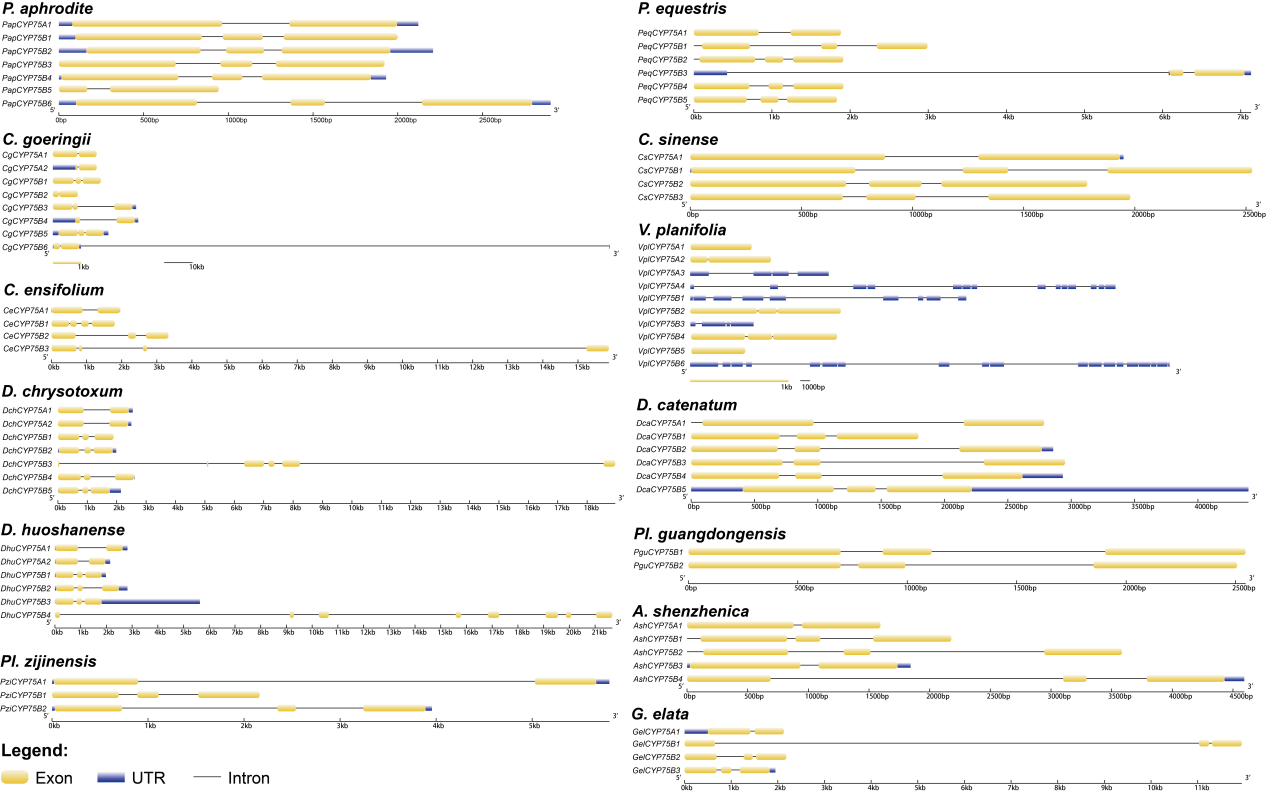
Figure S3. Gene structure of orchid *CYP75* genes.** Blue blocks, yellow blocks, and gray lines representing upstream or downstream-untranslated regions (UTR), exons, and introns, respectively.
